# Psychological skills and mindfulness for distance runners: a systematic review of performance, psychological outcomes, and implications for coaching practice

**DOI:** 10.3389/fpsyg.2026.1971379

**Published:** 2026-09-23

**Authors:** Wenhua Zhai, Qinghua Zhai, Ying Peng

**Affiliations:** 1 Geely University of China, Chengdu, China; 2 Moscow State University, Moscow, Russia; 3 Sichuan Vocational College of Finance and Economics, Chengdu, China

**Keywords:** athlete-centered coaching, coaching practice, distance running, mindfulness, performance, psychological skills training, self-regulation, sport psychology

## Abstract

**Background:**

Contemporary athlete support increasingly recognizes that performance preparation extends beyond physical training to the regulation of attention, effort, emotions, and behavior. Psychological skills training (PST) and mindfulness-based approaches offer different strategies for developing these capacities, yet their value for distance runners and their relevance to applied coaching remain uncertain. This systematic review examined runner-specific evidence for these interventions, distinguishing direct running performance from psychological and other performance-related outcomes.

**Methods:**

PubMed, Scopus, and Web of Science were searched from database inception to 2 April 2026. Eligible studies investigated PST or mindfulness-based interventions in distance runners using randomized, non-randomized comparative, or quasi-experimental designs. Risk of bias or methodological quality was evaluated using RoB 2, ROBINS-I, or the Joanna Briggs Institute checklist, according to study design. Substantial variation in runner populations, intervention approaches, comparators, assessment contexts, and outcome measures precluded quantitative pooling; therefore, evidence was synthesized narratively, with certainty considered using a narrative framework informed by GRADE principles.

**Results:**

From 28,602 records, 16 reports representing 15 unique evidence units met the eligibility criteria. Eleven evidence units examined PST and four investigated mindfulness-based interventions across middle-distance, cross-country, road, marathon, and ultramarathon settings. The clearest pattern concerned selected psychological outcomes rather than direct running performance. These psychological outcomes were not assessed uniformly: some were obtained in competition or running-specific contexts, others during laboratory running tasks, and others through broader sport-specific or dispositional questionnaires. Mindfulness-based interventions showed favorable signals for selected outcomes including flow, attentional refocusing, worry, and cognitive anxiety, whereas direct race or time-trial performance evidence was sparse and inconsistent. PST produced a more heterogeneous pattern. Some small, uncontrolled, or single-case studies reported favorable psychological or performance changes, but controlled comparisons did not demonstrate a consistent advantage. Confidence in these findings remained limited because of risk-of-bias concerns, small samples, heterogeneous outcomes, and the contribution of uncontrolled or single-case designs to several favorable findings.

**Conclusion:**

Psychological skills and mindfulness-based approaches may influence selected psychological processes relevant to running and competition, but current evidence does not establish that either approach reliably improves running performance. Implications for coaching remain provisional: the findings may inform cautious consideration of psychological strategies within broader athlete support, but they do not establish how coaches should integrate these interventions, whether individualized delivery is preferable, or which delivery approach is most effective.

**Systematic review registration:**

osf.io/gcpwy

## 1 Introduction

Distance-running performance emerges from the interaction of physiological capacity with the runner’s ability to regulate effort, pacing, attention, and psychological responses across training and competition (Midgley et al., 2007; McCormick et al., 2015; Sha et al., 2024). From an applied coaching and athlete-support perspective, these self-regulatory demands are particularly relevant because runners must continually adjust their behavior while coping with fatigue, discomfort, competitive pressure, and changing environmental conditions. Psychological preparation may therefore complement physical and technical training by targeting processes that influence how athletes interpret and respond to these demands. Psychological skills training (PST) typically involves the deliberate development of strategies such as goal setting, imagery, self-talk, relaxation, and attentional control to support performance and self-regulation (Birrer and Morgan, 2010; McCormick et al., 2015). Some performance-focused mental skills, such as routine goal setting, attentional cues, or structured self-talk, may be reinforced within coaching practice when they fall within the coach’s competence. This should be distinguished from structured psychological intervention and from assessment or treatment of clinically relevant psychological distress, for which appropriately qualified sport-psychology or mental-health expertise and, where necessary, referral are required (Carr, 2006; Bissett et al., 2020).

Mindfulness- and acceptance-based approaches take a different route, emphasizing present-moment awareness, acceptance, and reduced reactivity to internal experiences (Gardner and Moore, 2017; Röthlin et al., 2020; Wang et al., 2023). These approaches were combined at a higher analytical level because sport-specific mindfulness and acceptance programmes commonly include deliberate present-moment attention together with an open, non-judgmental or accepting relationship to thoughts, emotions, and bodily sensations. Acceptance-based models may additionally emphasize psychological flexibility, values, and committed action, and were therefore not considered conceptually identical to mindfulness training (Gardner and Moore, 2017). Thus, mindfulness and/or acceptance processes had to constitute an explicit and structured component of the intervention rather than occurring incidentally. Although conceptually distinct, both intervention families address psychological processes with potential relevance to athlete-centered preparation, including attentional regulation, perceived effort, anxiety management, coping with discomfort, and persistence under fatigue. Here, athlete-centered preparation refers to a needs-responsive and autonomy-supportive coaching process in which the runner’s perspective, preferences, personally meaningful goals, and response to previous practice inform intervention selection and adaptation, while the coach continues to provide appropriate structure and guidance (Mageau and Vallerand, 2003). This interpretation is aligned with autonomy-supportive coaching within Self-Determination Theory, where support for athlete autonomy, competence, and relatedness is combined with clear structure rather than equated with unrestricted athlete control (Raabe et al., 2019).

Their effects, however, may differ across psychological and performance outcomes, making it important not to assume that improvement in one domain necessarily translates into improvement in the other. PST is therefore used as an organizational umbrella rather than as a single uniform intervention. The individual techniques differ in their theoretical rationale, immediate psychological targets, and mode of application, and a multi-component PST programme should not be considered equivalent to an intervention using imagery, self-talk, goal setting, or another technique in isolation (Birrer and Morgan, 2010).

The rationale for these intervention families in endurance sport is grounded in established psychological determinants of endurance performance, including perception of effort, attentional focus, emotion regulation, and responses to stress and fatigue (Marcora et al., 2009; McCormick et al., 2015; Pageaux and Lepers, 2016). Running competitions also place substantial psychological demands on athletes, including the need to manage pacing decisions, competitive anxiety, pre-race stress, and fluctuations in concentration across prolonged efforts (Casado et al., 2021; Sha et al., 2024). Accordingly, outcomes of interest in this area extend beyond race performance to competition-related psychological constructs such as competitive anxiety, flow, coping, perceived stress, mindfulness, and emotion regulation (Dehghani et al., 2018; Wang et al., 2023). These outcomes also differ in their temporal and contextual level. A transient flow experience during a particular running task, for example, is conceptually different from a broader dispositional characteristic, while mindfulness and anxiety may themselves be measured as state-like or trait-like constructs depending on the instrument and assessment window (Jackson, 1996; Tanay and Bernstein, 2013). Thus, differences must be considered between state or task-/competition-specific outcomes and more dispositional or general outcomes when interpreting intervention effects, rather than treating change across these constructs as directly equivalent. Their responsiveness over interventions may also differ, so absence of change in a relatively stable construct should not be interpreted in the same way as absence of change in an immediately task-responsive state measure.

At the sport-wide level, systematic reviews and meta-analyses have reported generally favorable effects of psychological interventions on athletic performance and on several psychological outcomes, including benefits associated with imagery, broader PST approaches, and mindfulness-based interventions (Brown and Fletcher, 2017; Bühlmayer et al., 2017; Wang et al., 2023; Reinebo et al., 2024). However, these syntheses also emphasize important limitations, including substantial methodological heterogeneity, variable risk of bias, and limited certainty about which approaches are most effective within specific sports, populations, and outcome domains (Wang et al., 2023; Reinebo et al., 2024; Xie et al., 2025). Because sport-wide reviews pool athletes from sports with different performance constraints, task durations, psychological demands, and outcome definitions, their findings cannot be assumed to apply directly to distance runners. Within endurance sport specifically, prior evidence has suggested potential benefits of interventions such as imagery, self-talk, and goal setting, but the evidence base remains comparatively small and many studies have not been conducted in actual competition contexts (McCormick et al., 2015).

A runner-specific systematic review has already examined mindfulness practice in long-distance running. Such study (Corbally et al., 2020) reviewed mindfulness interventions in relation to mindfulness scores, physiological performance-related factors, psychological performance-related factors, and performance outcomes, identifying seven eligible studies and concluding that the evidence provided only tentative support because of methodological weaknesses. That review provided an important foundation for the mindfulness literature in runners, but it did not aim to synthesize the broader PST literature, synthesize PST and mindfulness-/acceptance-based evidence in parallel and contrast the resulting patterns descriptively. Thus, the specific gap addressed by the present review is not whether psychological interventions are generally beneficial in sport, but whether the runner-specific intervention literature provides credible evidence for objective performance effects, competition-related psychological effects, or both, and how certainty differs across intervention families and outcome domains.

The runner-specific intervention literature remains fragmented across study designs, intervention families, populations, and outcome definitions. Eligible studies include samples ranging from secondary-school middle-distance runners aged 13–16 years to collegiate, recreational, amateur, and high-level adult distance runners, and they examine interventions delivered through different formats, doses, comparators, and theoretical models. One included randomized trial specifically involved 28 female secondary-school runners aged 13–16 years (Donohue et al., 2006). Their inclusion introduces a developmental consideration that is less prominent in the adult running literature. Adolescence involves continuing cognitive and psychosocial development, while autonomy and self-regulation are also shaped by interactions with coaches, parents, and the wider youth-sport environment (McKay et al., 2016; Raabe et al., 2019; Bengtsson et al., 2024). Accordingly, the degree of independent practice, the language and complexity used to teach psychological strategies, and the involvement of coaches or parents may differ from adult delivery. Findings from adolescent runners are therefore interpreted within their developmental and support context rather than assumed to generalize directly to adult runners.

Against this background, the present systematic review provides an updated and broader synthesis of psychological interventions specifically examined in distance runners. Building on previous sport-wide evidence and the earlier runner-specific review of mindfulness (Corbally et al., 2020), we considered both PST and mindfulness-/acceptance-based approaches within a common framework while maintaining a clear distinction between objective performance outcomes and competition-related psychological outcomes. We also incorporated more recent runner-specific intervention studies and evaluated the evidence using design-appropriate risk-of-bias appraisal and narrative certainty-of-evidence assessment. Beyond determining whether favorable effects have been reported, the review was intended to establish how confidently such findings can inform applied practice. This distinction is particularly relevant for coaches and practitioners because changes in attention, anxiety, coping, flow, or other self-regulatory processes should not automatically be interpreted as evidence of improved running performance. Accordingly, the review aimed to synthesize in parallel the strength and consistency of the evidence for objective performance and competition-related psychological outcomes within PST and mindfulness-/acceptance-based intervention families, and to contrast the resulting patterns descriptively rather than estimate comparative effectiveness between intervention families. We also aimed to characterize the populations, intervention features, comparators, methodological limitations, and evidence gaps that constrain interpretation. Implications for coaching practice were derived interpretively from the intervention characteristics, participant contexts, provider information, outcome patterns, and certainty of the evidence; they were not treated as coaching outcomes directly tested by the included studies.

## 2 Methods

We conducted a completed systematic review of intervention studies examining whether psychological skills training and mindfulness-based interventions were associated with objective performance outcomes, competition-related psychological outcomes, or both, in distance runners. The review was structured in accordance with the Preferred Reporting Items for Systematic Reviews and Meta-Analyses (PRISMA 2020), and the reporting of electronic searching was aligned with relevant PRISMA-S guidance (Rethlefsen et al., 2021). The protocol was registered in the Open Science Framework on 31 March 2026 (ID: osf.io/gcpwy), before title and abstract screening commenced on the April 02, thus after the registration. The core review question, intervention families, outcome domains, and study-design eligibility criteria were not changed during the process.

### 2.1 Eligibility criteria

The eligibility criteria established *a priori* can be observed in Table 1. The criteria followed PICOS framework: participants, interventions, comparators, outcomes, and study design. In this review, distance running was used as an umbrella term for running-only competitive disciplines from middle-distance events to ultramarathon running, including 800 m, 1,500/1,600 m, cross-country, road running, marathon, and ultramarathon contexts. Middle-distance events were retained because they involve pacing, effort regulation, attentional control, and tolerance of fatigue that overlap with the psychological processes targeted by the eligible interventions; they were not assumed to be physiologically or psychologically equivalent to marathon or ultramarathon running.

**TABLE 1:** PICOS eligibility criteria.

PICOS element	Eligibility criterion
Participants	Distance runners participating in, or prospectively preparing for, an eligible running-only discipline, including middle-distance, long-distance, cross-country, road, marathon, and ultramarathon events. Mixed-sport samples were eligible only when runner-specific participant identity, intervention exposure or study condition, and at least one eligible outcome were separately extractable. Triathletes were not considered distance runners solely on the basis of the running component of triathlon. Participants not running at initial recruitment were eligible only when a subsequent prospective intervention phase explicitly involved preparation for and participation in an eligible distance-running event; only that phase contributed to the synthesis.
Interventions	Structured psychological skills training; mindfulness-based interventions; acceptance-based interventions; or hybrid approaches combining these elements. Mindfulness-/acceptance-based classification required an explicitly taught process involving present-moment attention and/or an accepting, non-judgmental relationship to internal experiences; acceptance-oriented programmes could additionally involve psychological flexibility, values, or committed action. Named programmes and their component structure were retained separately and were not assumed to be interchangeable. Acute priming, music, motivational prompting, brief yoga-only, or physical warm-up procedures were excluded unless they explicitly taught and evaluated a psychological skill or mindfulness-/acceptance-based process.
Comparators	No intervention, usual practice, wait-list, active comparator, alternative psychological intervention, or pre-intervention baseline in single-group or single-subject experimental designs.
Outcomes	Primary outcomes: direct running performance, defined as prospectively recorded race or standardized running time-trial performance, including time, speed or pace, race-derived splits or pacing, finishing position, or distance achieved. Other running-related outcomes: physiological, fitness, or laboratory indicators related to performance, including running economy, oxygen consumption, heart rate, ventilation, peak or intermittent running velocity obtained during standardized testing, and field or laboratory fitness-test performance; and behavioral participation/adherence outcomes, including starting or completing a target running event. Psychological outcomes: competition-related psychological constructs including competitive anxiety, pre-race stress, flow, coping, mindfulness, emotion regulation, attentional control, self-efficacy, perceived control, self-confidence, negative thoughts, or closely related constructs.
Study designs	Randomized trials, non-randomized controlled intervention studies, single-group quasi-experimental studies, and single-subject experimental designs with prospective intervention delivery and repeated outcome measurement. Observational studies without intervention, qualitative studies without outcome evaluation, case reports, reviews, editorials, protocols, and conference abstracts without sufficient data were excluded. Single-subject experimental designs were considered eligible when they prospectively delivered an intervention, included repeated outcome measurement across baseline and intervention phases, and allowed within-participant temporal comparison. These designs were distinguished from excluded case reports, which were defined as descriptive reports without a planned experimental intervention structure, repeated baseline/intervention measurement, or systematic outcome evaluation.
Other	No language restriction was applied. Non-English records were eligible and were translated as required for screening and full-text assessment. Publication status was not, in itself, an exclusion criterion provided that sufficient methodological and outcome information was available to establish eligibility and extract the required data. Full journal articles and other sufficiently detailed reports were eligible. Protocols, editorials, reviews, observational reports without an eligible intervention, qualitative reports without eligible outcome evaluation, descriptive case reports, and conference abstracts lacking sufficient methodological or outcome data were excluded.

For studies containing participants from more than one sport, inclusion required runner-specific data to be separable from those of non-running athletes. At minimum, the participant or subgroup had to be identifiable as a runner, the relevant intervention exposure or condition had to be distinguishable, and at least one eligible runner-specific outcome had to be extractable at the relevant assessment point. Triathletes were not classified as distance runners solely because running forms one component of triathlon. Where a mixed sample contained both triathletes and runners, only data attributable specifically to the runner or runner subgroup were retained. For studies with a staged design, population eligibility was applied at the beginning of the intervention phase contributing to the review rather than at the point of initial recruitment.

Participants who were not runners at initial recruitment could contribute only when a subsequent prospective study phase explicitly involved preparation for and participation in an eligible distance-running event. In such cases, only the intervention phase in which participants had committed to training for the running event, together with the corresponding running-related outcomes, was retained for the review.

### 2.2 Information sources

Electronic searches were conducted in PubMed, Scopus, and Web of Science Core Collection from database inception to 2 April 2026. These databases were selected to combine biomedical and exercise-science indexing through PubMed with broad multidisciplinary citation coverage through Scopus and Web of Science, including sport science, psychology, behavioral science, and rehabilitation journals relevant to psychological interventions in runners. We did not have institutional access to SPORTDiscus or PsycINFO. Therefore, these databases could not be searched directly. To mitigate this limitation, supplementary search procedures were completed after the database searches. These included backward citation searching of all included reports, backward citation searching of closely related systematic reviews and meta-analyses, forward citation searching of all included reports in Scopus and Web of Science, and specific verification searches using author names, intervention names, and outcome terms. Supplementary searches were completed on 2 April 2026, and all records identified through these procedures were exported, deduplicated, and screened using the same eligibility criteria as the database-derived records. After deduplication against the database records, 18 unique records identified through backward citation searching, forward citation searching, and targeted verification searches proceeded to title and abstract screening. All 18 were excluded at this stage, and no record proceeded to full-text assessment or contributed an additional included report.

### 2.3 Search strategy

The final search strategies were developed around two core concepts: (1) distance running and related competition formats; and (2) psychological skills training and mindfulness-based or acceptance-based interventions. Controlled vocabulary was used in PubMed where clearly relevant and available, and free-text synonyms, spelling variants, and sport-specific intervention terminology were added to improve retrieval of non-indexed and recently published studies.

The same two conceptual blocks (i.e., distance-running terms and psychological-intervention terms) were applied across all databases. Database-specific syntax was adapted to each platform: PubMed combined MeSH terms with title/abstract terms, Scopus used title/abstract/keyword fields, and Web of Science used topic-field searching.

The electronic searches were completed on 2 April 2026 using PubMed, Scopus, and Web of Science Core Collection via individual database platforms, with each source searched from database inception to the search date. No language restriction was applied, and no additional database filters or limits were used. Records retrieved from all sources were exported to EndNote online. Duplicate records were identified using EndNote deduplication procedure and were manually checked before removal. The deduplicated record set was then transferred to Excel for title and abstract screening and subsequent full-text assessment. Screening was undertaken independently by two authors using the predefined eligibility criteria, with disagreements resolved through discussion and, where necessary, consultation with a third author. No machine-learning, automated prioritization, or automated exclusion tools were used during study. Supplementary backward and forward citation searches and targeted verification searches were completed on the same date and were processed using the same record-management and eligibility procedures as the database-derived records.

PubMed:

((“Jogging”[Mesh] OR runner*[Title/Abstract] OR running [Title/Abstract] OR jogg*[Title/Abstract] OR “distance runner*”[Title/Abstract] OR “long-distance runner*”[Title/ Abstract] OR “middle-distance runner*”[Title/Abstract] OR “endurance runner*”[Title/Abstract] OR marathon*[Title/ Abstract] OR “half marathon*”[Title/Abstract] OR “half-marathon*”[Title/Abstract] OR ultramarathon* [Title/ Abstract] OR ultrarunning[Title/Abstract] OR “ultra-running”[Title/Abstract] OR “cross-country”[Title/Abstract] OR “cross country”[Title/Abstract] OR “road run*”[Title/Abstract]))

AND

((“psychological skills training”[Title/Abstract] OR “mental skills training”[Title/Abstract] OR “mental training”[Title/Abstract] OR imagery[Title/Abstract] OR “mental imagery”[Title/Abstract] OR visuali*[Title/Abstract] OR “self-talk”[Title/Abstract] OR “self talk”[Title/Abstract] OR “goal setting”[Title/Abstract] OR relaxation[Title/Abstract] OR “attention control”[Title/Abstract] OR “arousal regulation”[Title/Abstract] OR “emotional control”[Title/Abstract] OR “cognitive restructuring”[Title/Abstract] OR mindful*[Title/Abstract] OR “Mindfulness”[Mesh] OR meditat* [Title/Abstract] OR “mindfulness-based”[Title/Abstract] OR “mindful sport performance enhancement”[Title/Abstract] OR MSPE[Title/Abstract] OR “mindfulness-acceptance-commitment”[Title/Abstract] OR “mindfulness acceptance commitment”[Title/Abstract] OR “acceptance and commitment”[Title/Abstract] OR “acceptance-based”[Title/ Abstract] OR “Acceptance and Commitment Therapy”[Mesh]))

Scopus:

TITLE-ABS-KEY(runner* OR running OR jogg* OR “distance runner*” OR “long-distance runner*” OR “middle-distance runner*” OR “endurance runner*” OR marathon* OR “half marathon*” OR “half-marathon*” OR ultramarathon* OR ultrarunning OR “ultra-running” OR “cross-country” OR “cross country” OR “road run*”)

AND

TITLE-ABS-KEY(“psychological skills training” OR “mental skills training” OR “mental training” OR imagery OR “mental imagery” OR visuali* OR “self-talk” OR “self talk” OR “goal setting” OR relaxation OR “attention control” OR “arousal regulation” OR “emotional control” OR “cognitive restructuring” OR mindful* OR meditat* OR “mindfulness-based” OR “mindful sport performance enhancement” OR MSPE OR “mindfulness-acceptance-commitment” OR “mindfulness acceptance commitment” OR “acceptance and commitment” OR “acceptance-based”)

Web of Science Core Collection:

TS = (runner* OR running OR jogg* OR “distance runner*” OR “long-distance runner*” OR “middle-distance runner*” OR “endurance runner*” OR marathon* OR “half marathon*” OR “half-marathon*” OR ultramarathon* OR ultrarunning OR “ultra-running” OR “cross-country” OR “cross country” OR “road run*”)

AND

TS = (“psychological skills training” OR “mental skills training” OR “mental training” OR imagery OR “mental imagery” OR visuali* OR “self-talk” OR “self talk” OR “goal setting” OR relaxation OR “attention control” OR “arousal regulation” OR “emotional control” OR “cognitive restructuring” OR mindful* OR meditat* OR “mindfulness-based” OR “mindful sport performance enhancement” OR MSPE OR “mindfulness-acceptance-commitment” OR “mindfulness acceptance commitment” OR “acceptance and commitment” OR “acceptance-based”)

Search results from all databases were exported to Endnote, and duplicate records were removed before screening.

### 2.4 Selection process

After deduplication, title and abstract screening was performed independently by two authors. Neither the search nor study eligibility was restricted by publication language. Non-English records were assessed on the same eligibility criteria as English-language records. Where the title or abstract was not available in a language understood by the reviewers, its content was translated sufficiently to permit eligibility assessment. Full texts considered potentially eligible were similarly translated as required before a final inclusion or exclusion decision was made. The full texts of potentially eligible articles were then assessed independently by the same authors against the predefined eligibility criteria. Disagreements were resolved first through discussion and, if necessary, by consultation with a third author. Reasons for full-text exclusion were recorded.

### 2.5 Data collection process

Data extraction was undertaken independently by two authors using a piloted standardized extraction form. Any discrepancies were reconciled by consensus or adjudication by a third author. Where outcome data or intervention details were incompletely reported, attempts were made to derive the necessary information from the article text, tables, appendices, or supplementary files. For study accounting, we distinguished records, reports, unique studies, and evidence units. A report was defined as an individual publication or article. A unique study was defined as a distinct participant sample, intervention, comparator, and study design. An evidence unit was defined as the unique study-level dataset contributing eligible outcomes to the synthesis. When multiple companion reports described the same underlying study, they were linked and counted once in study-level and evidence-unit summaries, while all eligible outcomes from the companion reports were extracted under the same evidence unit (Page et al., 2021).

### 2.6 Data items and outcomes

The following study-level variables were extracted: bibliographic details; country; study design; setting; sample size; age; sex; competitive level; running discipline; inclusion and exclusion criteria; intervention type and theoretical framework; intervention components; session frequency, duration, and total dose; delivery format; provider characteristics where reported; attendance and adherence to the prescribed intervention; completion of home-based or independent practice; any reported assessment of intervention fidelity; facilitator training, qualifications, or competence; deviations from the planned intervention; contamination between intervention and comparator conditions; and relevant psychological, behavioral, or training co-interventions occurring during the study period; comparator characteristics; outcome measures; follow-up timing; adverse events or unintended effects if reported; and funding source or conflicts of interest where available. Implementation variables were extracted only when they could be established from the primary report or accompanying supplementary material. When an implementation characteristic was not described, it was recorded as not reported rather than interpreted as having been adequately controlled or as not having occurred.

Running-related outcomes were classified into three distinct domains before synthesis. Direct running performance comprised prospectively recorded performance from an actual race or a standardized running time trial, including completion time, running speed or pace, split or pacing data derived from the performance itself, finishing position, or distance achieved under a defined task. Physiological, fitness, or laboratory indicators related to performance were retained separately and included measures such as running economy, oxygen consumption, heart rate, ventilation, peak or intermittent running velocity obtained during laboratory or field fitness testing, and standardized shuttle- or fitness-test outcomes. These measures were considered relevant to performance but were not interpreted as direct evidence of improved race or time-trial performance. Participation or adherence outcomes comprised behavioral endpoints such as starting or completing a target event or maintaining participation in the prescribed running goal when these were reported as study outcomes. Intervention attendance, home practice, and compliance with the psychological programme remained implementation variables and were not treated as participation outcomes. Competition-related psychological outcomes were retained as a separate fourth domain. Race-derived pacing variables were classified as direct running performance when they represented objectively recorded pace, speed, or split behavior during an eligible race or time trial. By contrast, self-reported use of pacing, drafting, self-talk, imagery, or other strategies was classified according to the relevant psychological or behavioral construct rather than as running performance.

Where reported, the role and professional background of the intervention provider were extracted and considered as characteristics of intervention delivery. Provider roles were distinguished as coach, sport-psychology or mental-health practitioner, researcher/interventionist, or other/unclear according to the information given in the primary report. Any intervention-specific training, supervision, or fidelity procedures reported for the facilitator were also retained. Practitioner competence was not inferred when qualifications were not described, and evidence from a researcher- or specialist-delivered programme was not assumed to establish that the same intervention could be delivered independently by a coach.

The timing of outcome assessment was retained because intervention effects could plausibly differ between an initial post-intervention response and effects maintained after further practice or competition exposure. For the narrative synthesis, assessments were therefore classified using operational follow-up windows: immediate post-intervention, defined as assessment at intervention completion or within 7 days; short-term follow-up, defined as more than 7 days and up to 6 weeks after intervention completion; and longer-term follow-up, defined as more than 6 weeks after intervention completion. These intervals were used as descriptive categories rather than as assumptions about the expected duration of an intervention effect. Where more than one assessment was available within the same window, the assessment closest to the end of the intervention was used to represent that period.

For outcomes linked to a particular running task or competition, the assessment associated with that task or event was retained even when its timing did not coincide exactly with the general follow-up windows. This applied separately to direct running-performance measures, event participation/completion outcomes, and task-specific psychological outcomes such as flow. Immediate and later assessments were not treated as interchangeable.

For psychological outcomes, the construct and assessment context were also retained to distinguish state or task-/competition-specific measures from more dispositional or general measures where this distinction was supported by the instrument and timing of assessment. These categories were used for interpretation rather than as additional pooled outcome groups. Psychological outcome measures were additionally classified according to their measurement basis. Validated sport-specific measures were defined as standardized instruments for which published measurement-property evidence was available in sport, exercise, or athlete populations and whose items were framed specifically around sport or performance. Validated general measures comprised established standardized instruments with published measurement-property evidence but without a specifically sport-based measurement context. Bespoke or unvalidated measures included study-developed items, author-generated ratings or indices, modified measures for which an appropriate validation basis could not be identified, and other psychological outcomes reported without sufficient evidence to establish measurement validity. Where a general instrument had simply been administered to athletes, it remained classified as a general measure unless sport-specific validation evidence was available. Classification was based on the instrument described in the primary report and its cited measurement sources rather than on the direction or statistical significance of the study findings. We did not undertake a separate formal psychometric appraisal of each instrument. Event initiation or completion was retained when reported prospectively within an eligible running intervention, but was classified as a behavioral participation or adherence outcome rather than as direct running performance.

### 2.7 Risk of bias assessment

Randomized trials were appraised with RoB 2 (Sterne et al., 2019). For each randomized trial, the target result was the principal eligible result contributing to the narrative synthesis for the outcome shown in results. Where available, this was the post-intervention between-group contrast or group-by-time result most directly aligned with the review outcome. When a trial contributed more than one eligible outcome, separate RoB 2 assessments were undertaken when the target results differed sufficiently in missing-data, outcome-measurement, or selective-reporting considerations to warrant outcome-specific judgment.

Non-randomized comparative intervention studies were appraised with ROBINS-I (Sterne et al., 2016). Single-group and single-subject quasi-experimental studies were appraised with the JBI critical appraisal tool for quasi-experimental studies (Barker et al., 2024). Risk-of-bias and critical-appraisal assessments were conducted independently by two reviewers, with disagreements resolved through discussion or consultation with a third reviewer. These judgments were carried forward into the synthesis and used to qualify the interpretation of individual findings.

For the JBI appraisals, item-level responses were retained as the primary assessment. Moderate methodological confidence was assigned where temporal ordering was clear and the study retained an interpretable repeated-measures or pre/post structure, with generally complete follow-up and an analysis considered appropriate for that design, despite important limitations such as absence of a concurrent control or uncertainty in some measurement items. Low methodological confidence was assigned where several limitations affected the intervention comparison simultaneously, particularly when a concurrent control and repeated pre/post assessment were absent and follow-up, measurement reliability, or analytical approach introduced additional uncertainty. No quasi-experimental study was classified as high methodological confidence because all retained important design limitations.

### 2.8 Effect measures

For continuous outcomes reported on the same scale across studies, mean differences with 95% confidence intervals were retained. When conceptually similar continuous outcomes were measured using different instruments, standardized mean differences with small-sample correction were used. For dichotomous outcomes, risk ratios or odds ratios with 95% confidence intervals were used if such data were encountered. Effect measures were extracted as reported by the primary studies or derived when sufficient numerical information was available. For continuous outcomes, we retained means, standard deviations, mean differences, standardized effects, and 95% confidence intervals where these were reported or could be calculated. For dichotomous outcomes, we extracted event counts and reported relative-effect estimates where available. When the required numerical data were unavailable, findings were summarized descriptively. These effect measures were used to structure the narrative synthesis and to describe the direction and magnitude of findings where reported or calculable. They were not pooled because the included studies did not provide sufficiently comparable combinations of population, intervention family, comparator, and outcome definition.

### 2.9 Synthesis methods

Because the included studies differed in runner population, running discipline, intervention content, comparator condition, study design, outcome definition, and measurement approach, quantitative pooling was not considered appropriate. The synthesis was therefore conducted without meta-analysis, with reporting structured in accordance with the Synthesis Without Meta-analysis (SWiM) guideline (Campbell et al., 2020).

Studies were first organized by intervention family: psychological skills training, mindfulness-/acceptance-based intervention, or hybrid intervention. Within the psychological skills training group, the identity of the intervention was retained, so that single-technique approaches such as self-talk, imagery, or goal setting were not treated as equivalent to multi-component programmes. Within each intervention family, findings were considered separately across four outcome domains: (1) direct running performance; (2) physiological, fitness, or laboratory indicators related to performance; (3) participation or adherence outcomes; and (4) competition-related psychological outcomes. An evidence unit could contribute to more than one domain when it reported distinct eligible outcomes. The domains were therefore not treated as mutually exclusive study categories. When several running-related outcomes were available, direct race or time-trial performance was given greater interpretive weight than physiological, fitness, or laboratory indicators. Participation or event-completion outcomes were interpreted separately and were not used to support claims of improved running performance.

Direction of effect was determined at the outcome level rather than by assigning a single direction to the study as a whole. In controlled studies, the between-group contrast or group-by-time interaction was given priority over within-group change when both were available. Within-group results were retained as contextual information but were not interpreted as evidence that the intervention was superior to the comparator. For single-group and single-case studies, change from the relevant pre-intervention or baseline phase to the intervention or post-intervention phase was retained. When a study reported several eligible outcomes within the same domain, individual results were preserved. Findings were described as favorable when the retained comparative estimate or inferential result supported the intervention in the intended direction, comparator-favoring when the direction favored the comparator, no clear advantage when no meaningful comparative difference was demonstrated, and mixed when eligible outcomes within the same domain showed different directions. Statistical significance was considered together with effect magnitude, precision where available, comparator characteristics, study design, and risk of bias rather than used as the sole basis for classification.

When several results addressed the same review question, interpretive priority was given to outcomes most directly aligned with that question. For objective performance, race time, time-trial performance, running speed or pace, finishing position, distance achieved, or another direct measure of running performance was given greater interpretive weight than laboratory or physiological proxies. Event initiation or completion without a standardized performance metric was treated separately as a behavioral participation or adherence outcome and was not included in the direct performance synthesis. For psychological outcomes measuring a similar construct, sport- or task-specific validated measures were given greater interpretive weight than general or bespoke measures, while all eligible results remained available in the outcome table. Assessment timing followed the operational windows described in Section “2.6 Data items and outcomes,” with immediate and later observations interpreted separately rather than combined.

Heterogeneity was examined descriptively across runner age and competitive level, running discipline, intervention components and dose, delivery and implementation characteristics, comparator type, outcome directness and measurement basis, assessment timing, study design, and risk of bias. No statistical measure of heterogeneity was calculated because no pooled synthesis was undertaken.

Descriptive sensitivity analyses were conducted by repeating the interpretation after setting aside, in turn: (1) runner-specific extracts from mixed-sport samples; (2) the study in which participants were not runners at initial recruitment; (3) randomized trials judged at high risk of bias; and (4) uncontrolled or single-subject studies. These analyses were used to assess whether the direction or strength of the review conclusions depended on these methodologically distinct subsets. No effect estimates were recalculated or pooled for the sensitivity analyses.

### 2.10 Narrative certainty assessment informed by GRADE principles

Because the evidence could not be combined quantitatively and the available studies were sparse and methodologically heterogeneous, we did not conduct a formal outcome-specific GRADE assessment with prespecified starting levels and sequential downgrading or upgrading decisions. Instead, confidence in each intervention-family/outcome-domain synthesis was evaluated narratively using the main considerations represented in the GRADE framework. These included risk of bias, consistency of findings, directness of the evidence to distance-running performance or competition-related psychological outcomes, and imprecision arising from small samples or unstable estimates. Study design, comparator quality, outcome measurement, and the availability and precision of effect estimates were considered when applying these judgments.

Potential reporting bias was also considered conceptually, but it could not be assessed formally because the number of studies within each intervention-family/outcome-domain group was small, study designs and outcomes were heterogeneous, and there were too few comparable estimates for funnel-plot or statistical assessment. Selective outcome reporting within individual randomized or non-randomized studies was considered within the relevant risk-of-bias tool rather than treated as a formal body-level assessment of publication bias. The resulting low and very-low ratings in results are therefore narrative certainty descriptors informed by GRADE principles rather than formal GRADE ratings derived from an outcome-specific evidence profile.

## 3 Results

### 3.1 Study selection

A total of 28,602 records were identified through database searching, comprising 1,883 from PubMed, 9,554 from Scopus, and 17,165 from Web of Science. After removal of 7,410 duplicates, 21,192 unique records underwent title and abstract screening. The large reduction from title/abstract screening to full-text assessment reflected the intentionally sensitive search strategy, which retrieved many records concerning running, exercise, mindfulness, meditation, or psychological constructs that did not evaluate a structured PST or mindfulness-based intervention in distance runners. Most records excluded at title/abstract screening were therefore removed because they were not runner-specific intervention studies, did not involve an eligible psychological skills training or mindfulness-based intervention, or did not report eligible objective performance or competition-related psychological outcomes.

Twenty-two reports were assessed in full text. Six reports were excluded at the full-text stage: four because they did not contribute an eligible retained outcome (Thompson et al., 2011; Samadi et al., 2020; Basset et al., 2022; Grandjean da Costa et al., 2022), and two because the intervention did not meet the operational definition of psychological skills training or mindfulness-based intervention (Donohue et al., 2006; Goddard et al., 2024). Basset et al. (2022) was excluded because the study examined physiological responses and perceived exertion during a fixed-intensity running task without an eligible objective running-performance or competition-related psychological outcome. Grandjean da Costa et al. (2022) was excluded because the retained outcomes were affective valence, arousal, and perceived exertion during a fixed-intensity laboratory run rather than an eligible performance or competition-related psychological endpoint. Thompson et al. (2011) was excluded because runner performance at follow-up was reported retrospectively through a questionnaire rather than as an objectively measured performance endpoint, while the main psychological follow-up analyses combined runners with athletes from other sports. Samadi et al. (2020) was excluded because the study assessed general cognitive emotion-regulation strategies and salivary cortisol rather than an eligible competition-related psychological or objective running-performance outcome. Donohue et al. (2006) was excluded because the brief motivational-shouting and yoga procedures did not meet the operational definition of structured psychological skills training or a mindfulness-/acceptance-based intervention. Goddard et al. (2024) was excluded because the intervention manipulated open goals, performance feedback, and metronome tempo to facilitate flow rather than explicitly teaching an eligible psychological skill or mindfulness-/acceptance-based process.

Sixteen reports were included in the review. These reports represented 15 unique evidence units because two companion journal articles (Barbry et al., 2025a) reported different outcome domains from the same underlying mindfulness-based randomized trial and were therefore linked for study-level accounting. Barbry et al. (2025b) contributed objective performance-related and cardiorespiratory-fitness outcomes, whereas Barbry et al. (2025a) contributed sport-specific mindfulness and well-being outcomes. Figure 1 presents the study-selection flow, with the final inclusion reported at both report and unique-study/evidence-unit levels. Supplementary search procedures identified no additional eligible report.

**FIGURE 1:**
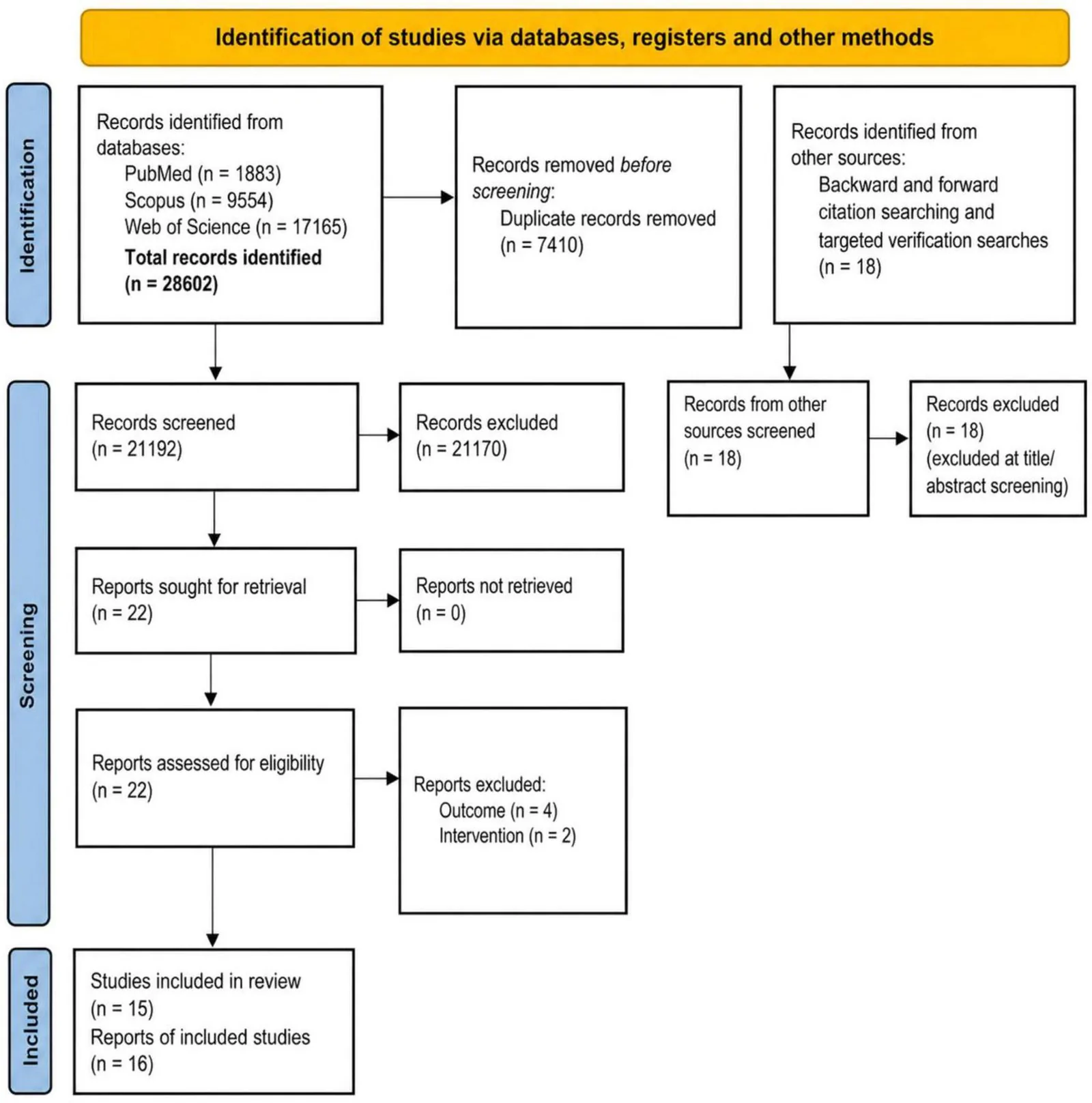
Preferred Reporting Items for Systematic Reviews and Meta-Analyses (PRISMA) flow diagram showing database and supplementary-source identification, duplicate removal, title and abstract screening, full-text assessment, exclusion reasons, and final inclusion. Sixteen reports representing 15 unique studies/evidence units were included in the review.

### 3.2 Characteristics of included studies

Findings were summarized by intervention family, outcome domain, direction of effect, and methodological confidence. Seven unique evidence units contributed at least one direct running-performance outcome. Three contributed physiological, fitness, or laboratory indicators related to performance, and one contributed an event-participation/completion outcome. Nine evidence units contributed at least one core competition-related psychological outcome. These categories were not mutually exclusive because individual studies could contribute findings to more than one domain. Direct running-performance outcomes included official race or time-trial time, running speed or pace, 800-m, 1,600-m, one-mile, 2.3-km and 5-km performance, and objectively recorded race-pacing data. Performance-related indicators included running economy, oxygen consumption, heart rate, ventilation, peak or intermittent running velocity obtained during standardized testing, and shuttle- or fitness-test outcomes. The characteristics of the included unique evidence units are summarized in Table 2.

**TABLE 2:** Characteristics of included reports and unique evidence units.

References	Participants/context	Design/comparator	Intervention and dose	Implementation/provider	Assessment timing	Implementation/provider
Barbry et al., 2025a,b	65 trained/highly trained French Athletics Federation runners were randomized (18-65 y); 55 were retained in the psychological companion analysis after attrition.	Randomized parallel-group trial. Brief mindfulness meditation (BMM) + common HIIT running programme versus active running-information control + the same HIIT programme.	FeelTheRun, 8 wk. Three brief in-person BMM contacts/wk (about 5–12 min), delivered before/after training and later integrated into running; optional home audio practice. Control: weekly running-information sessions, with one progressive-muscle-relaxation session.	Programme developed/delivered by investigators with sport-psychology and mindfulness expertise. Weekly self-report of BMM frequency captured in-person + autonomous practice; audio/app support and twice-weekly videoconference access were available. Coaches standardized the common HIIT programme.	Baseline, 4 wk, and 8 wk for sport-mindfulness measures; baseline and 8 wk for well-being/fitness outcomes. No later follow-up.	Attendance/adherence: weekly self-report of total mindfulness sessions; exact attendance at scheduled supervised contacts not reported. Home practice: autonomous audio/video practice permitted and included in weekly session count; separate home-practice completion not reported. Fidelity: formal mindfulness-intervention fidelity assessment not reported; common HIIT programme standardized by coaches. Provider: investigators with sport-psychology/mindfulness expertise; sport-psychology input reported in programme development/implementation.
Caird et al., 1999	7 competitive male and female long-distance runners.	Single-group time-series quasi-experimental study: 6-wk usual-training control phase followed by 6-wk intervention phase; participants served as their own controls.	Three laboratory contacts/wk for 6 wk. Supervised progressive muscular relaxation and centering plus treadmill biofeedback; centering prescribed for 15 min/day at home using heart-rate feedback.	Relaxation practice and usual training were recorded in logs. Sessions were supervised, but facilitator qualifications were not specified. Regular training/racing continued throughout.	Pre-control, post-control (6 wk), and post-intervention (12 wk from baseline).	Attendance/adherence: relaxation practice and usual training recorded in logs; laboratory-session attendance rate not reported. Home practice: centering prescribed for 15 min/day; completion rate not reported. Fidelity: not reported. Provider: sessions supervised; professional qualifications not reported.
Cooper et al., 2021	Original sample: three experienced female Masters endurance athletes. Only P3, a 40-years-old experienced trail runner and high-school track coach, met the review’s runner-specific population criterion; P1 and P2 were triathletes and were not included in the synthesis.	Non-concurrent single-subject multiple-baseline design. Runner-specific evidence uses P3’s own baseline, intervention, and follow-up phases.	Personalized strategic self-talk using motivational and instructional cue words/phrases, adjusted iteratively from participant feedback. Four intervention 800-m trials followed by three follow-up trials without prompts.	Researcher-led and observed at the track. No formal home-practice programme was reported. Intervention exposure was directly observed during intervention trials; follow-up sessions removed researcher-provided self-talk prompts.	Repeated 800-m trials across about 10 wk; P3 completed 6 baseline, 4 intervention, and 3 follow-up trials.	Attendance/adherence: eligible runner completed 6 baseline, 4 intervention, and 3 follow-up trials. Home practice: not applicable; no formal independent practice component prescribed. Fidelity: intervention exposure directly observed during intervention trials; no separate formal fidelity rating. Provider: researcher-led; professional qualifications not reported.
Cardenas Hernandez et al., 2025	6 recreational long-distance runners (5 men, 1 woman), 26-35 y, with mean running experience about 5.5 y.	Single-subject A-B-A exploratory design. First official race served as pre-intervention comparison; second official race followed VR training.	Approx. 1-h education + 1–2-h device onboarding, then ≥9 home-based VR sessions over 3 wk (15–20 min/session) targeting imagery, self-talk, pacing, drafting, and motivation.	Participants trained independently at home; VR log files provided exposure data (one participant had missing device logs but completed questionnaires/races). Usual physical training continued and was not standardized. Race distances (5–10 km) differed between assessments/participants.	Race 1 pre-intervention; repeated assessments around each VR session; Race 2 after the 3-wk VR phase (whole study about 4 wk).	Attendance/adherence: at least 9 VR sessions prescribed; exposure recorded from device logs, with log data missing for one participant. Home practice: intervention completed independently at home. Fidelity: formal assessment not reported. Provider: education and device onboarding were provided; professional qualifications not reported.
Hill et al., 2021	31 moderately trained runners (13 men, 18 women); mindfulness *n* = 16, wait-list *n* = 15.	Randomized wait-list controlled trial. Control participants maintained their usual sport routine during the comparison period.	8-wk mindfulness programme. Daily independent mindfulness practice was required; after 2 wk, participants were instructed to incorporate mindfulness into running and refocus non-judgmentally on a self-selected movement component.	Mindfulness diaries documented activity, duration, and perceived quality. A telephone contact after 2 wk reviewed progress. Diaries were returned at post-test. Facilitator qualifications were not clearly reported.	Pre-intervention and immediate post-intervention laboratory testing at 8 wk.	Attendance/adherence: mindfulness diaries and a 2-weeks telephone review were used; quantitative adherence rate not reported. Home practice: daily mindfulness practice prescribed and recorded in diaries; completion rate not reported. Fidelity: formal assessment not reported. Provider: professional qualifications not reported.
Jaenes et al., 2021	14 amateur male marathon runners (21-40 y; mean about 30 y), with substantial running/marathon experience.	Single-group pre-post quasi-experimental study; no concurrent control because participants were receiving psychological support at the centre.	7-wk cognitive-behavioral PST; weekly 60–90-min sessions. First 2 sessions group-based, remaining sessions individual. Techniques included association/dissociation, thought stopping/self-talk, progressive relaxation, coordination/tactical skills, and individualized adjustment.	Delivered and supervised by a sport psychologist. Progressive relaxation prescribed daily; skills practiced at home and during running. The psychologist continuously adjusted application while preserving the programme structure.	Baseline and post-intervention assessment; final post-intervention psychological scale was collected 48 h before the target marathon.	Attendance/adherence: seven weekly sessions prescribed; attendance rate not reported. Home practice: daily relaxation and application of skills during running prescribed; completion not reported. Fidelity: no formal fidelity measure reported. Provider: sport psychologist.
Kelemen et al., 2025	20 Hungarian national/international distance runners (800 m-marathon); MSPE *n* = 10, control *n* = 10.	Non-randomized controlled pre-post study. Control received no organized sport-psychology programme.	Runner-adapted MSPE for 6 wk; weekly 90-min online group sessions. Formal/informal mindfulness, running meditation, 3R practice, and daily homework.	Led by a trained MSPE instructor with an MSc in Athletics Coaching/long-distance specialization. Homework journals were reviewed weekly; feedback/reminders and a satisfaction survey supported adherence. Other mental training was reported by 3 intervention and 2 control athletes, although none had prior mindfulness/meditation experience.	Pre-test and post-test 1 wk after the final MSPE session.	Attendance/adherence: weekly homework journals reviewed with feedback/reminders; session-attendance rate not reported. Home practice: daily homework prescribed; quantitative completion rate not reported. Fidelity: formal fidelity assessment not reported. Provider: trained MSPE instructor with an MSc in Athletics Coaching/long-distance specialization.
Koehn and Díaz-Ocejo, 2022	3 male junior middle-distance runners, 14–18 y, competing nationally in Qatar and internationally on training camps.	Non-concurrent single-case A-B multiple-baseline design; each athlete served as own control.	Individualized imagery script incorporating relaxation and performance-specific imagery; 10–15 min at home, 3 times/wk for 4 wk.	Sport psychologist co-developed and introduced scripts with coach input; a practice session preceded independent use. Adherence logbooks tracked session date/duration and imagery experience. Important co-intervention: the same sport psychologist was already delivering weekly general psychological-skills work to the athletes during the season.	Approx. 4-wk baseline (6–9 trials), 4-wk imagery intervention, and 4-wk post-intervention phase (7–8 trials).	Attendance/adherence: all three participants completed three imagery sessions/week and at least 12 sessions over 4 weeks. Home practice: home-based imagery; mean recorded session duration approximately 17 min. Fidelity: introductory practice session and adherence log used; no separate fidelity rating. Provider: sport-psychology involvement with coach input; concurrent general psychological-skills support also occurred.
McCormick et al., 2018	32 ultramarathon entrants randomized; 29 attended the event and entered analyses (self-talk *n* = 14, concentration-grid control *n* = 15).	Randomized parallel-group field trial during a 60-mile overnight ultramarathon; active concentration-grid comparator.	Individual intake plus workbook intervention. Self-talk group identified/refined motivational statements and practiced them in training until race day; active control practiced concentration grids. Four practice logs were requested in each condition.	Primary researcher conducted standardized intake. Compliance was checked before the event: 12/14 self-talk participants reported using the workbook and 10 submitted logs; 12/15 controls reported concentration-grid practice and 10 submitted logs. No participant reported being told the other workbook’s content.	Pre-event assessments, official race outcome, immediate post-event surveys, and 6-mo follow-up of continued strategy use.	Attendance/adherence: 12/14 self-talk participants reported workbook use and 10 returned logs; 12/15 controls reported concentration-grid practice and 10 returned logs. Home practice: both groups practiced their assigned strategy before the ultramarathon. Fidelity: standardized intake and contamination check; no formal fidelity score. Provider: primary researcher; professional qualifications not reported.
Patrick and Hrycaiko, 1998	Original sample: one elite male 1,500-m runner and three male triathletes. Only the elite runner (P3) met the review’s runner-specific population criterion and contributed to the synthesis; the three triathletes were excluded from the extracted runner-specific result.	Single-subject multiple-baseline design. P3’s own baseline and intervention data provide the runner-specific evidence.	Mental-skills package: relaxation, imagery, self-talk, and goal setting. Taught individually over 3 d; first 2 sessions about 90 min, followed by workbook-based practice.	Daily researcher contact checked homework. Workbook exercises/report sheets and a standardized behavioral checklist were used as procedural-fidelity checks. Mental-skills use was measured after each trial as a manipulation check.	Repeated baseline and intervention 1,600-m trials; no distinct longer-term follow-up for the retained runner.	Attendance/adherence: workbook exercises were completed and homework was checked through daily researcher contact; runner-specific mental-skills use was monitored across trials. Home practice: workbook-based practice. Fidelity: behavioral checklist, procedural-reliability procedures, and manipulation check used. Provider: researcher-delivered; professional qualifications not sufficiently reported.
De Petrillo et al., 2009	25 recreational long-distance runners (10 men, 15 women), 18–55 y; MSPE *n* = 13 and waiting-list *n* = 12.	Open, non-randomized waiting-list controlled trial; some participants selected workshop timing because of scheduling constraints.	Runner-adapted MSPE, 4 weekly sessions of 2.5–3 h. Body scan, sitting meditation, mindful yoga, walking/running meditation, and application to running.	Led by the first author. Between sessions, participants used guided-audio material, practiced mindfulness during running, and kept practice/running logs. In the initial MSPE group, 9/13 attended all 4 sessions, 3 attended 3, and 1 withdrew after session 1.	Baseline and immediate post-workshop (4 wk). Six-month practice/running logs were requested, but later follow-up was not used as a core retained outcome in this evidence unit.	Attendance/adherence: 9/13 attended all four sessions, 3/13 attended three, and 1/13 withdrew after the first session. Home practice: guided audio, mindfulness practice during running, and practice logs; completed home-practice dose not reported. Fidelity: formal assessment not reported. Provider: first author; professional qualifications not sufficiently reported.
Rhodes et al., 2021	Stage 1: 31 self-described non-runners received motivational interviewing. Five months later, 15 who agreed to train for/attempt an ultramarathon entered Stage 2 (FIT *n* = 7; comparison *n* = 8).	Stage-2 randomized pilot comparison after a shared motivational-interviewing lead-in.	FIT: group-based functional imagery around goals, obstacles, multisensory imagery, self-talk cues, and coping; comparison: performance/training discussion without specific imagery. Booster contact about 2 mo before the event and an email the day before.	Initial MI was delivered by a trained, experienced practitioner with reported high fidelity. Participants managed their own running programmes; researchers did not prescribe physical training. Race distance and conditions varied. FIT-specific facilitator credentials/fidelity were not clearly reported.	Stage-2 questionnaires before intervention and after the race; target races occurred later in the training period (50 km to >100 mi, depending on participant).	Attendance/adherence: 15 participants entered Stage 2; uptake of subsequent booster contacts not reported. Home practice: self-directed running and cue-based imagery practice were incorporated; completion not reported. Fidelity: initial motivational interviewing reported as high-fidelity; FIT-specific fidelity not reported. Provider: initial motivational interviewing delivered by a trained, experienced practitioner; FIT-provider qualifications not reported.
Robinson et al., 2023	7 experienced competitive parkrun/5-km runners (4 men, 3 women), 29-45 y; intervention *n* = 4, active control *n* = 3.	Small randomized mixed-method field study with extensive historical/repeated within-participant performance data.	Brief online self-help videos delivered sequentially: if-then planning (44 s), self-talk (41 s), and reappraisal (44 s). Active control was encouraged to mentally prepare using previously established strategies.	Standardized videos reduced provider variation; no therapist contact was required. Post-run questionnaires assessed whether strategies were used. All runners continued usual training. The active control itself encouraged psychological self-regulation, limiting separation of specific intervention effects from study/expectancy effects.	Two-months historical baseline plus repeated 5-km assessments over about 12 wk (baseline, if-then, self-talk, reappraisal, and post-study data).	Attendance/adherence: strategy use assessed through post-run questionnaires; quantitative adherence rate not reported. Home practice: runners were encouraged to use the skills in training/racing; completion not reported. Fidelity: standardized prerecorded videos; no separate fidelity assessment. Provider: self-help delivery without therapist contact.
Tenenbaum et al., 1999	28 female secondary-school middle-distance runners, 13–16 y.	Blocked randomized allocation to 3 active goal-difficulty conditions: easy, difficult/realistic, or improbable/unattainable. No no-goal control.	Weekly short- and long-term performance goals over 4 wk; goals were individualized from baseline running time and reiterated before each weekly 2.3-km run.	Goals were delivered individually; athletes verbally accepted/committed to them. A manipulation check assessed perceived goal difficulty/attainment. Participants were asked not to share assigned goals. No home-practice or fidelity procedure beyond goal delivery/checks was reported.	Baseline plus weekly 2.3-km runs for 4 wk.	Attendance/adherence: attendance rate not reported; athletes verbally accepted their assigned goals and were reminded of them before each run. Home practice: not applicable; no independent practice component prescribed. Fidelity: goal-difficulty manipulation check conducted; participants were asked not to disclose assigned goals. Provider: experimenter; professional qualifications not reported.
Weinberg et al., 2012	81 collegiate cross-country runners (41 men, 40 women), 18–24 y, from 6 teams.	Six-arm matched/yoked experimental comparison; allocation was based on baseline mile time and yoking, not randomization. All groups received an active self-talk condition.	Motivational, instructional, or combined self-talk × self-selected or assigned statements. Personalized 3-min audio script was listened to about 5 min before the post-test mile.	Single standardized audio exposure; no requirement to use statements during the run. Coaches timed/encouraged both trials and reported similar training loads. A post-experimental questionnaire assessed reactions; no independent fidelity or contamination assessment was reported.	Baseline mile and post-test about 8 d later.	Attendance/adherence: single standardized pre-run exposure; adherence to self-talk during the mile itself was not directly verified. Home practice: not applicable. Fidelity: prerecorded 3-min audio standardized the intervention; post-experimental reactions were collected, but no direct fidelity check was reported. Provider: psychological content delivered by standardized audio; coaches timed and encouraged the running trials.

Barbry et al. (2025a,b) were treated as two reports but one unique evidence unit because they reported outcomes from the same randomized trial. “Not clearly reported” should not be interpreted as evidence that fidelity was adequate or that contamination/co-intervention did not occur. HIIT, high-intensity interval training; MSPE, Mindful Sport Performance Enhancement; PST, psychological skills training; VR, virtual reality.

Outcome assessment was concentrated around intervention completion or the first relevant post-intervention running task or competition. Later follow-up was less consistently available, limiting comparison of immediate effects with the persistence or consolidation of change over time.

Table 3 summarizes the retained findings by intervention family and the four prespecified outcome domains. Directional labels describe the reported direction of findings and should not be interpreted as evidence of established effectiveness. Findings from uncontrolled, single-case, high-risk, or serious-risk studies were interpreted in light of their methodological limitations and the narrative certainty assessment. Figure 2 maps the distribution of evidence across direct running performance, physiological, fitness, or laboratory indicators related to performance, participation or adherence outcomes, and competition-related psychological outcomes. Direct race or standardized time-trial evidence was confined to PST studies, whereas mindfulness-based studies contributed performance-related physiological or fitness indicators and psychological outcomes but no direct race or standardized time-trial outcomes. Figure 2 maps these outcome domains separately and shows that direct running-performance evidence was confined to PST studies, whereas mindfulness-based studies contributed psychological outcomes and performance-related physiological or fitness indicators but no direct race or time-trial evidence.

**TABLE 3:** Direction, quantitative support, and methodological confidence of retained findings by report and outcome domain.

References	Outcome measure(s)	Outcome domain	Measurement classification	Timing	Main findings	Interpretation for synthesis
Barbry et al., 2025a	Intermittent maximum speed and 3-min shuttle-run test (plus HRV, RPE, body composition/fitness variables).	PFI	OBJ - direct test-based/physiological measures; no psychometric classification applicable.	Baseline, 4 wk and/or 8 wk, depending on outcome (immediate post-intervention at 8 wk).	Time x group interaction for cardiorespiratory fitness tests, including intermittent maximum speed and 3-min shuttle run; control declined while BMM remained comparatively stable (reported *p* = 0.01). Other outcomes showed no clear interaction.	Favorable but indirect performance-related signal; does not establish improved race performance.
Barbry et al., 2025b	Mindfulness Inventory for Sport (MIS): awareness, acceptance, refocusing. Diagnofeel: daily-life well-being manifestation/regulation.	PSY	MIS = VSSM. Diagnofeel = VGM (general well-being, not sport-specific).	MIS: baseline, 4 wk, 8 wk. Diagnofeel: baseline and 8 wk.	Refocusing: time × group *p* = 0.03, eta-squared = 0.07; BMM 3.40 ± 0.85 to 3.82 ± 0.67; control 3.33 ± 0.94 to 3.28 ± 0.79; between-group at 8 wk *p* = 0.01. No clear group advantage for awareness or acceptance. Selected Diagnofeel subscales also favored BMM.	Core competition-related signal is sport-specific refocusing. General well-being findings should be kept separate from competition-specific outcomes.
Caird et al., 1999	Peak running velocity; oxygen uptake, heart rate, and ventilation at lactate threshold.	PFI	OBJ - performance/physiological measures.	Pre-control, post-control (6 wk), post-intervention (12 wk from baseline).	Peak running velocity remained 18.29 ± 1.98 km/h across assessments. From post-control to post-intervention, VO2 at lactate threshold decreased 48.77 ± 3.85 to 45.44 ± 4.02 mL/kg/min; HR 161.86 ± 11.26 to 157.86 ± 11.72 bpm; ventilation 86.05 ± 16.09 to 78.80 ± 17.42 L/min.	Physiological-economy signal without direct improvement in peak running velocity.
Cooper et al., 2021	800-m finish time; Mental Toughness Index (MTI; wording adapted to the 800-m context). RPE, urge to slow, and perceived performance were ancillary.	DRP + PSY	Finish time = OBJ. MTI = VGM, context-adapted; the modified wording was not independently revalidated in this study. Ancillary perceived-performance ratings were bespoke.	Repeated baseline, intervention, and follow-up trials over ∼10 wk.	For the eligible runner (P3), the standardized mean difference was 3.7 for 800-m finish time and 1.6 for Mental Toughness Index score. Finish time improved by more than 17 s from the final baseline trial to the first intervention trial, and mean Mental Toughness Index scores were higher during the intervention phase. Results for P1 and P2 were not retained because both participants were triathletes.	Favorable single-case finding in one eligible runner. The result should not be generalized to the full three-participant sample because the other two participants did not meet the prespecified runner definition.
Cardenas Hernandez et al., 2025	Smartwatch-derived race/pacing metrics; Emotional Stress Reaction Questionnaire (ESRQ); modified Post-Race Questionnaire (PRQ) assessing motivation, self-talk, imagery, concentration, confidence, commitment, pacing/drafting, and open-ended responses.	DRP + PSY	Race metrics = OBJ. PRQ = BU (modified/study-specific). ESRQ = BU/insufficiently validated for this specific use because measurement-property evidence was not established in the primary report.	Race 1 pre-intervention; repeated pre/post VR-session ESRQ; Race 2 after 3 wk VR training.	Mean race appraisal-index difference changed from −0.33 ± 5.41 (Race 1) to 2.50 ± 1.50 (Race 2). Most participants reported greater use of self-talk, imagery, concentration, pacing/drafting strategies; smartwatch data suggested more stable pacing for some individuals. Formal causal inference was limited.	Exploratory descriptive signal. Changes in race distance/context and reliance on modified self-report tools substantially limit interpretation.
Hill et al., 2021	Flow State Scale-2 (FSS-2); Five Facet Mindfulness Questionnaire-Short Form (FFMQ-SF); oxygen consumption/running economy.	PFI + PSY	FSS-2 = VSSM (state/task-specific). FFMQ-SF = VGM (dispositional/general). Oxygen consumption = OBJ.	Pre-intervention and immediate post-intervention at 8 wk.	Flow showed a group × time interaction (*F* = 26.13, *p* < 0.001, partial eta-squared = 0.40) and increased within the mindfulness group. Oxygen consumption improved within the mindfulness group (*F* = 4.99, *p* = 0.044, partial eta-squared = 0.28), but the group × time interaction was not significant. Dispositional mindfulness did not clearly change.	Stronger signal for task-specific flow than for running economy; no direct race-performance endpoint.
Jaenes et al., 2021	Eight-item mental-state scale covering negative thoughts before/during the marathon and mental load/self-doubt after the race; items partly derived from the Spanish CSAI-2R.	PSY	BU - study-created, partially adapted measure; internal consistency reported (alpha = 0.690), but no separate validation of the final 8-item instrument.	Baseline and after the 7-wk intervention; final post-intervention scale collected 48 h before the target marathon.	Negative-thought scores decreased for pre-race items (*Z* = −3.316, *p* < 0.05, *d* = 2.385) and during-race items (*Z* = −3.305, *p* < 0.05, *d* = 3.051); mental load/self-doubt items also decreased (*Z* = −3.307, *p* < 0.05, *d* = 2.731).	Large within-group changes on a bespoke measure; no concurrent control, so findings are hypothesis-generating.
Kelemen et al., 2025	Hungarian Flow State Questionnaire (FSQ); Competitive State Anxiety Inventory-2 Hungarian version (CSAI-2H); Mindful Attention Awareness Scale-Hungarian (MAAS-H); State Difficulties in Emotion Regulation Scale (S-DERS).	PSY	FSQ = VGM/state-flow measure. CSAI-2H = VSSM. MAAS-H and S-DERS = VGM.	Pre-test and post-test 1 wk after the 6-wk intervention (immediate window).	Flow: group x time *F*(1,18) = 12.45, *p* = 0.002, partial eta-squared = 0.41. Cognitive anxiety: *F* = 10.534, *p* = 0.004, partial eta-squared = 0.37. Mindfulness: *F* = 6.212, *p* = 0.02, partial eta-squared = 0.26. Somatic anxiety: *F* = 3.69, *p* = 0.07. Emotion-regulation difficulties did not show a clear interaction.	Favorable comparative signal for selected psychological outcomes, but non-random allocation and other mental-training exposure remain important limitations.
Koehn and Díaz-Ocejo, 2022	Flow State Scale-2 Short Form (FSS-2 SF), Arabic translation prepared by back-translation.	PSY	VSSM; the underlying instrument is validated for sport/task flow, but the Arabic translation used here did not undergo a separate full psychometric validation in this study.	Repeated baseline (∼4 wk), 4-wk intervention, and 4-wk post-intervention phase.	Mean flow increased for all 3 athletes by about 1.43-3.13 points; binomial tests *p* < 0.01 for each; reported non-overlap effect sizes 0.63, 0.88, and 0.57.	Replicated single-case flow signal, interpreted cautiously because of sample size and concurrent season-long psychological support.
McCormick et al., 2018	Official ultramarathon finish time/completion; task-specific self-efficacy and perceived-control scales designed for the study. Sport Emotion Questionnaire and other state ratings were ancillary.	DRP + PA + PSY	Performance = OBJ. Self-efficacy/perceived control = BU (task-specific scales constructed for this study following Bandura guidance). Sport Emotion Questionnaire = VSSM (ancillary).	Pre-event, during event, immediate post-event; 6 mo follow-up assessed continued strategy use rather than a durable treatment effect on performance.	Finishers within advertised cut-off: self-talk 824 ± 97 min vs. control 836 ± 75 min, *t*(18) = 0.31, *p* = 0.76, *d* = 0.13. Including all finishers: 853 ± 114 vs. 848 ± 75 min, *p* = 0.90, *d* = −0.05. No condition x time effect for self-efficacy or perceived control.	No clear performance or pre-event psychological advantage despite acceptable intervention use; long-term follow-up supports continued use, not efficacy.
Patrick and Hrycaiko, 1998	1,600-m running time; study-specific mental-skills-use questionnaire used as a manipulation check.	DRP	Running time = OBJ. Mental-skills-use questionnaire = BU/ manipulation check.	Repeated baseline and intervention trials; no distinct longer-term retained follow-up.	For the eligible runner (P3), mean 1,600-m performance improved by 8.25 s from the baseline phase to the intervention phase. Mental-skills use increased from 28.86% during baseline to 100% during intervention. Results from the three triathletes were not included in the review synthesis.	Favorable single-case performance finding in one eligible elite runner. The finding is based on within-participant change and should not be interpreted as evidence from the complete four-athlete sample.
De Petrillo et al., 2009	Sport Anxiety Scale (SAS); Thought Occurrence Questionnaire for Sport (TOQS); Multidimensional Perfectionism Scale (MPS); Kentucky Inventory of Mindfulness Skills (KIMS); Toronto Mindfulness Scale (TMS); self-reported running logs.	PSY; self-reported running behavior ancillary	SAS and TOQS = VSSM. MPS, KIMS, and TMS = VGM. Running logs = OBJ/self-reported performance data. Credibility/expectancy and background items were study-adapted/bespoke ancillary measures.	Baseline and immediate post-workshop at 4 wk.	Organizational demands improved more than wait-list control [*F*(1,19) = 6.20, *p* < 0.03]. Among workshop completers, sport-anxiety worry fell 15.57–13.09 (*t* = 2.35, *p* < 0.05); acting with awareness rose 25.10–28.30 (*t* = 4.33, *p* < 0.05); state mindfulness rose 29.00–34.38 (*t* = 2.30, *p* < 0.05). Best mile time, running distance, and frequency did not improve.	Selected psychological changes were favorable; objective running performance was unchanged. Open/non-random allocation limits causal attribution.
Rhodes et al., 2021	Official ultramarathon start/completion; Short Grit Scale (Grit-S); General Self-Efficacy Scale (GSE); Brief Resilience Scale (BRS); Plymouth Sensory Imagery Questionnaire (Psi-Q).	PA; general psychological measures ancillary	Event start/completion = behavioral participation and event-adherence outcome; not classified as a direct objective performance measure. Grit-S, GSE, BRS, and Psi-Q = validated general psychological measures.	Stage-2 baseline before group intervention and post-race; race timing varied by participant. No standardized short/long follow-up window across events.	FIT: 7/7 started and 6/7 finished; comparison: 4/8 started and 2/8 finished. OR = 18 (95% CI 1.27–255.76), *p* = 0.033; RR = 5.25; Fisher exact *p* = 0.0406. No significant group/time differences on the self-report scales.	Favorable signal for event participation and completion rather than direct running performance. The estimate was highly imprecise and arose from a small sample of participants who were initially non-runners and managed their own physical training.
Robinson et al., 2023	Official parkrun/5-km performance cross-checked with Strava; post-run questions on preparation, strategy use, perceived effectiveness, and experience.	DRP; ancillary study-specific psychological/strategy measures	5-km performance = OBJ. Post-run psychological/strategy-use questions = BU.	Historical 2-mo baseline plus repeated assessments across ∼12 wk; each brief skill was evaluated at a separate period.	Overall performance differed by condition/time [*F*(4,83) = 2.78, *p* = 0.03, partial eta-squared = 0.12]. Intervention observations averaged 4.57 ± 0.40 m/s versus 4.75 ± 0.26 m/s in active control. Relative to pre-study data, active control improved by 347.37 m and intervention by 164.11 m; *post hoc p* = 0.019 favored active-control improvement over baseline.	No evidence that the specific videos outperformed active mental-preparation encouragement; strong expectancy/self-regulation effects are plausible.
Tenenbaum et al., 1999	2.3-km running time. Task and Ego Orientation in Sport Questionnaire (TEOSQ) was measured before intervention as a dispositional moderator; end-study goal-difficulty manipulation check.	DRP	Running time = OBJ. TEOSQ = VSSM but not an intervention outcome. Goal-difficulty manipulation check = BU.	Baseline plus weekly runs for 4 wk.	All conditions improved over time; whole sample improved 5.23% at wk 4. Goal-difficulty groups did not differ significantly in improvement. Personal-best improvement averaged 7.82% [*F*(3,75) = 3.61, *p* < 0.02].	Performance improved during the study, but no specific goal-difficulty prescription showed superiority.
Weinberg et al., 2012	One-mile running time. Self-talk preference ratings and post-experimental reaction questionnaire were ancillary study-specific measures.	DRP	Running time = OBJ. Ancillary self-talk/reaction ratings = BU.	Baseline and post-test about 8 d later.	Overall time improved from 5:32 ± 0:52 to 5:25 ± 0:48; trial effect *F*(1,75) = 44.67, *p* < 0.001, partial eta-squared = 0.37. No significant main effect of self-talk type or assigned versus self-selected delivery. Condition × trial interaction *F*(5,75) = 2.24, *p* = 0.05, partial eta-squared = 0.13.	Short-term improvement occurred, but matched/yoked allocation and absence of a no-self-talk control prevent a randomized causal interpretation or identification of a clearly superior self-talk format.

OBJ, objective performance or physiological measure; VSSM, validated sport-specific measure; VGM, validated general measure; BU, bespoke, modified, insufficiently validated, or study-specific measure. DRP, direct running performance; PFI, physiological, fitness, or laboratory indicator related to performance; PA, participation/adherence outcome; PSY, competition-related psychological outcome; VSSM, validated sport-specific measure; VGM, validated general measure; BU, bespoke, modified, or insufficiently validated measure.

**FIGURE 2:**
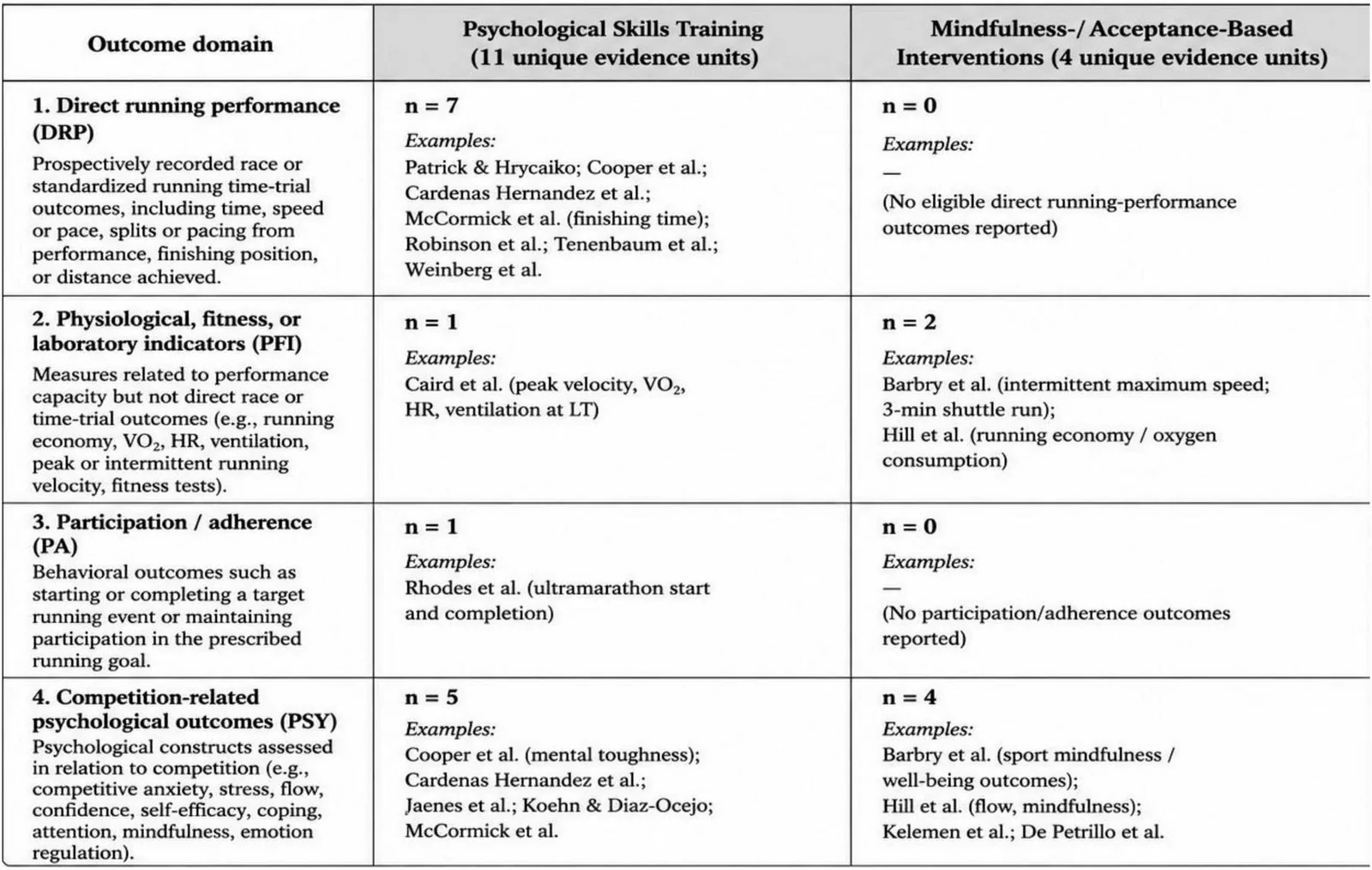
Evidence map by intervention family and outcome domain, counted at the unique-evidence-unit level.

#### 3.2.1 Intervention implementation and delivery

Reporting of intervention implementation varied considerably across the included studies (Table 2). Several reports monitored exposure to the psychological intervention, although quantitative attendance or adherence was not consistently provided. In the Barbry et al. (2025b), runners reported each week how many brief mindfulness sessions they had completed, combining supervised and autonomous practice. Hill et al. (2021) used mindfulness diaries to record the type, duration, and perceived quality of practice and included a telephone contact after 2 weeks. Kelemen et al. (2025) reviewed homework journals weekly and provided reminders and feedback. Koehn and Díaz-Ocejo (2022) reported the clearest home-practice data: all three runners recorded three imagery sessions per week and completed at least 12 sessions over 4 weeks, with mean session durations of approximately 17 min. De Petrillo et al. (2009) also provided attendance data; nine of 13 participants attended all four Mindful Sport Performance Enhancement sessions, three attended three sessions, and one withdrew after the first session. In the ultramarathon study by McCormick et al. (2018), 12 of 14 runners allocated to self-talk reported using the workbook and 10 returned practice logs; in the active-control group, 12 of 15 reported practicing concentration grids and 10 returned logs. Where the reports described monitoring procedures but did not provide a numerical completion rate, adherence was recorded as not reported rather than assumed to be satisfactory.

Independent practice formed part of several interventions but was also unevenly documented. Caird et al. (1999) prescribed 15 min of daily centering practice and used participant logs, while Jaenes et al. (2021) prescribed daily progressive relaxation and encouraged application of the psychological skills during running. The Barbry et al. (2025b) programme allowed autonomous practice using audio or video material, and De Petrillo et al. (2009) supplied guided recordings and asked runners to practice mindfulness between workshops and during running. Cardenas Hernandez et al. (2025) delivered the intervention largely at home and used virtual-reality log files to record exposure, although device data were unavailable for one participant. In McCormick et al. (2018), both study groups were asked to practice their allocated strategy before race day. Robinson et al. (2023) encouraged runners to trial the brief psychological skills in training and assessed subsequent strategy use by questionnaire. Where home practice was prescribed but its completion was not quantified, this was recorded as not reported. Home practice was considered not applicable when no independent practice component was prescribed, rather than being classified as missing.

Patrick and Hrycaiko (1998) used workbook records, daily researcher contact, a standardized behavioral checklist, and a post-run manipulation check to examine whether the mental-skills package was being implemented as planned. In Cooper et al. (2021), intervention exposure was directly observed during the 800-m trials, and the runner completed six baseline, four intervention, and three follow-up trials. Koehn and Díaz-Ocejo (2022) used adherence logs and an introductory practice session before independent imagery practice. Tenenbaum et al. (1999) reiterated the assigned goals before each run and used an end-of-study manipulation check; participants were also asked not to disclose their assigned goals to other runners. Rhodes et al. (2021) reported that the initial motivational-interviewing phase was delivered by a trained and experienced practitioner with high fidelity, although fidelity of the subsequent Functional Imagery Training component was not reported. Robinson et al. (2023) used standardized prerecorded videos, which limited variation in intervention delivery, but no separate fidelity assessment was reported. For the remaining studies, a formal fidelity assessment was not reported.

Information about the intervention provider was similarly incomplete. Jaenes et al. (2021) identified a sport psychologist as the intervention provider, Kelemen et al. (2025) reported delivery by a trained Mindful Sport Performance Enhancement instructor with postgraduate training in athletics coaching and long-distance running, and Koehn and Díaz-Ocejo (2022) described sport-psychology involvement together with coach input. The Barbry et al. (2025b) programme was developed and implemented with sport-psychology and mindfulness expertise. Rhodes et al. (2021) described the practitioner delivering the initial motivational-interviewing phase as trained and experienced, but the qualifications of the provider responsible for the Functional Imagery Training phase were not sufficiently specified. Several other reports identified a researcher or experimenter as the person delivering or supervising the intervention but did not provide enough professional information to judge practitioner competence; these cases were recorded as provider qualifications not reported. Overall, the implementation evidence was sufficient to establish that many interventions involved repeated practice and some form of exposure monitoring, but it was generally insufficient to determine whether intervention dose and fidelity were comparable across studies. This limits interpretation of both favorable and null findings, particularly where an absence of effect cannot be separated from incomplete exposure or uncertain delivery fidelity.

#### 3.2.2 Harms and unintended effects

Reporting of harms and unintended effects was limited across the included studies. None of the reports described a prospective, structured procedure specifically designed to monitor adverse events attributable to the psychological intervention. In most studies, intervention-related harms or unintended effects were not reported. Cooper et al. (2021) described the strategic self-talk intervention as not being accompanied by side effects, although no formal adverse-event monitoring procedure was reported. De Petrillo et al. (2009) reported that one participant withdrew from the mindfulness intervention after the first session because of health problems; the report did not attribute those problems to the intervention. McCormick et al. (2018) documented pain, injury, blisters, navigation difficulties, fatigue, and adverse weather during the ultramarathon, but these were described as demands or stressors associated with the event rather than harms caused by the self-talk intervention.

### 3.3 Risk of bias and critical appraisal within included studies

Risk-of-bias and critical-appraisal findings are presented in Tables 4–6. Six unique randomized trials contributed seven RoB 2 target-result assessments because the Barbry et al. trial contributed separate performance-related and psychological target results. Five of the seven target-result assessments were judged at high risk of bias and two as having some concerns. At the unique-trial level, four trials had at least one target result judged at high risk of bias, whereas two had only some-concerns judgments. The most recurrent problems were incompletely reported allocation concealment, lack of preregistration or prespecified analysis plans, outcome-specific missing data or selective retention of completers, and reliance on self-reported psychological outcomes obtained from unblinded participants. Three non-randomized comparative studies were evaluated with ROBINS-I, and all three were judged at serious risk of bias, principally because intervention allocation was non-random and some outcomes relied on self-report from unblinded participants. Among the six quasi-experimental or single-case studies appraised with the Joanna Briggs Institute checklist, five were rated as having moderate methodological confidence and one as having low methodological confidence. Although several of these studies benefited from repeated within-participant measurements and clear temporal ordering, the absence of concurrent controls, continued routine training, very small samples, and limited psychometric information for some outcomes constrained causal interpretation. These risk-of-bias and critical-appraisal findings were carried forward into the narrative synthesis and certainty judgments, so favorable findings from high-risk, serious-risk, uncontrolled, or single-subject designs were interpreted as low- or very-low-certainty signals rather than as evidence of established effectiveness.

**TABLE 4:** Risk of bias judgments for randomized trials assessed with RoB 2.

References	Target result	Randomization process	Deviations from intended interventions	Missing outcome data	Measurement of outcome	Selection of reported result	Overall risk of bias
Barbry et al., 2025b	MIS refocusing at 8 weeks (time-by-group/post-intervention contrast)	Some concerns	Some concerns	High	High	Some concerns	High
Barbry et al., 2025a	Objective cardiorespiratory-performance result at 8 weeks (intermittent maximum speed/3-min shuttle-run test)	Some concerns	Some concerns	High	Some concerns	Some concerns	High
Hill et al., 2021	Flow during the post-intervention laboratory running task	Some concerns	Some concerns	Low	High	Some concerns	High
McCormick et al., 2018	Official ultramarathon finishing time	Some concerns	Some concerns	High	Low	Some concerns	High
Rhodes et al., 2021	Official ultramarathon start/completion status	Some concerns	Some concerns	Low	Low	Some concerns	Some concerns
Robinson et al., 2023	Official 5-km/parkrun performance	Some concerns	Some concerns	Low	Low	High	High
Tenenbaum et al., 1999	2.3-km running performance across goal-difficulty conditions	Some concerns	Some concerns	Low	Low	Some concerns	Some concerns

MIS, Mindfulness Inventory for Sport.

**TABLE 5:** Risk of bias judgments for non-randomized controlled studies assessed with ROBINS-I.

References	Target result	Confounding	Selection of participants	Classification of interventions	Deviations from intended interventions	Missing data	Measurement of outcomes	Selection of reported result	Overall risk of bias
Kelemen et al., 2025	Flow and cognitive-anxiety group-by-time results after MSPE	Serious	Moderate	Low	Moderate	Low	Serious	Moderate	Serious
De Petrillo et al., 2009	Sport-anxiety worry and related post-intervention psychological outcomes	Serious	Moderate	Low	Moderate	Moderate	Serious	Moderate	Serious
Weinberg et al., 2012	One-mile performance across self-talk conditions (condition-by-trial result)	Serious	Low	Low	Moderate	Low	Low	Moderate	Serious

ROBINS-I, Risk Of Bias In Non-randomized Studies - of Interventions; MSPE, Mindful Sport Performance Enhancement.

**TABLE 6:** Critical appraisal judgments for quasi-experimental and single-case studies assessed with the Joanna Briggs Institute checklist.

References	Outcome	Cause/ effect clear	Participants similar	Treatment similar apart from intervention	Control group	Multiple pre/post measure ments	Follow-up complete	Measured same way	Measured reliably	Appropriate analysis	Review-level methodo logical confidence
Caird et al., 1999	Objective performance (peak running velocity)	Yes	Yes	Unclear	No	Yes	Yes	Yes	Yes	Yes	Moderate
Cooper et al., 2021	Runner-only 800 m finish time and mental toughness	Yes	Yes	Unclear	No	Yes	Yes	Yes	Yes	Yes	Moderate
Cardenas Hernandez et al., 2025	Race-strategy adoption, psychological outcomes, and pacing-related performance	Yes	Yes	No	No	No	No	Yes	Unclear	No	Low
Jaenes et al., 2021	Race-related negative thoughts, mental load, and self-doubt	Yes	Yes	No	No	No	Yes	Yes	Unclear	Yes	Moderate
Koehn and Díaz-Ocejo, 2022	Flow during the running task	Yes	Yes	Unclear	No	Yes	Yes	Yes	Unclear	Yes	Moderate
Patrick and Hrycaiko, 1998	Runner-only objective 1,600-m time	Yes	Yes	Unclear	No	Yes	Yes	Yes	Yes	Yes	Moderate

JBI, Joanna Briggs Institute.

### 3.4 Results of individual studies

The findings were synthesized narratively, first by intervention family and then by outcome domain. Table 3 summarizes the retained findings at unique evidence-unit level, with companion reports linked under the same evidence unit where applicable.

#### 3.4.1 Psychological skills training interventions

Evidence for direct objective performance outcomes following PST was mixed. Favorable within-participant changes were observed in the runner-specific single-case evidence from Patrick and Hrycaiko (1998) and Cooper et al. (2021). In Patrick and Hrycaiko (1998), only the elite 1,500-m runner was eligible for the present synthesis, and his mean 1,600-m time improved by 8.25 s after introduction of the mental-skills package. In Cooper et al. (2021), only P3, the trail runner, was retained; her 800-m finish-time result favored the intervention phase relative to baseline. These findings arose from individual single-case data and were not taken from the triathletes included in the original reports.

Rhodes et al. (2021) was considered separately because its principal behavioral outcome was whether participants started and completed an ultramarathon rather than a direct measure of running performance. In Stage 2, all seven participants allocated to Functional Imagery Training started an ultramarathon and six completed it, compared with four starters and two finishers among eight participants in the comparison group. This finding was treated as a participation/completion signal and not as evidence of faster or otherwise improved running performance.

Among studies contributing direct performance outcomes, Cardenas Hernandez et al. (2025) reported exploratory changes in pacing-related variables, whereas McCormick et al. (2018) did not identify a significant advantage of motivational self-talk over an active control for ultramarathon finishing time. Robinson et al. (2023) found greater improvement in the active-control mental-preparation condition than in the pooled intervention condition. Tenenbaum et al. (1999) and Weinberg et al. (2012) reported improvement over time, but neither study established a clear advantage of one goal-setting or self-talk condition over the alternatives.

Performance-related physiological or laboratory evidence within the PST literature was limited to Caird et al. (1999). Peak running velocity did not change across the study phases, whereas oxygen uptake, heart rate, and ventilation at lactate threshold decreased after the relaxation and biofeedback intervention. These observations were treated as physiological or running-economy signals rather than evidence of improved direct running performance.

#### 3.4.2 Mindfulness-based interventions

No mindfulness-based study contributed a prospectively recorded direct race or standardized running time-trial outcome. Performance-related evidence was limited to physiological, fitness, or laboratory indicators. Barbry et al. (2025a) reported favorable time-by-group interactions for intermittent maximum speed and the 3-min shuttle-run test, whereas Hill et al. (2021) reported a within-group reduction in oxygen consumption during running without a clear between-group interaction. These findings were retained as performance-related physiological or fitness signals and were not interpreted as evidence that mindfulness improved race performance.

The psychological evidence for mindfulness-based interventions was more consistently positive, but remained low certainty. Barbry et al. (2025b) reported improved sport-specific refocusing, whereas awareness and acceptance did not show clear between-group differences. Hill et al. (2021) found that flow during the laboratory running task improved after the 8-weeks mindfulness program. Kelemen et al. (2025) reported improvement in flow and cognitive anxiety relative to the control condition, whereas somatic anxiety did not show a clear between-group advantage. De Petrillo et al. (2009) found improvements in competition-related worry and cognitive interference after Mindful Sport Performance Enhancement, although interpretation was limited by open allocation and the absence of randomization.

#### 3.4.3 Sensitivity analyses

The descriptive sensitivity analyses did not change the overall interpretation of the evidence. Setting aside the runner-specific extracts from the mixed-sport studies by Patrick and Hrycaiko (1998) and Cooper et al. (2021) removed two favorable single-case performance findings, but the remaining comparative evidence still did not show a consistent benefit of PST for direct running performance. Excluding Rhodes et al. (2021), whose participants were not runners at initial recruitment, removed the favorable participation/completion signal but did not alter the direct-performance synthesis because event start and completion were analyzed separately from running performance.

When randomized trials judged at high risk of bias were set aside, the remaining randomized evidence was too sparse to support a consistent intervention effect. The favorable psychological findings from some mindfulness studies were reduced, while the remaining randomized evidence still did not establish a reliable performance benefit. Finally, removing uncontrolled and single-subject studies substantially reduced the favorable evidence for psychological skills training because several of its positive performance and psychological findings arose from these designs. Among the remaining comparative studies, objective performance findings remained inconsistent, whereas selected mindfulness-related psychological outcomes continued to show favorable signals in some studies. Overall, the sensitivity analyses strengthened rather than weakened the need for cautious interpretation and did not change the main conclusion that the evidence is more suggestive for selected psychological outcomes than for reliable improvement in running performance.

### 3.5 Certainty of evidence

Narrative certainty judgments informed by GRADE principles are summarized in Table 7. Confidence remained low or very low across all intervention-family/outcome-domain combinations. These ratings are descriptive certainty judgments rather than formal GRADE evidence profiles. Formal assessment of reporting bias was not possible because the number of studies contributing to each synthesis group was small and the outcome and design structure was too heterogeneous for meaningful statistical assessment.

**TABLE 7:** Narrative certainty of evidence by intervention family and outcome domain.

Intervention family	Outcome domain	Evidence pattern	Main considerations informing narrative certainty	Narrative certainty
Psychological skills training	Objective performance outcomes	Direct performance findings were inconsistent. Favorable signals were concentrated in small single-case studies and the ultramarathon pilot, whereas comparative studies commonly showed no clear advantage for the focal strategy over an active comparator or over alternative psychological conditions. Setting aside mixed-sport extracts, the initially non-running sample, and uncontrolled or single-case evidence further weakened the favorable pattern.	Frequent risk-of-bias concerns; substantial variation in event distance, intervention content, comparator, and performance endpoint; some indirect or context-dependent outcomes; small samples and unstable estimates; favorable findings were sensitive to exclusion of weaker designs.	Very low
Mindfulness-based interventions	Objective performance or performance-related outcomes	Evidence was sparse and largely indirect. Barbry et al. reported favorable time-by-group effects for fitness-test outcomes, while Hill et al. reported a within-group change in running economy without a clear comparative effect. Direct race-performance evidence was absent.	High or substantial risk-of-bias concerns in key studies; indirectness because available outcomes were laboratory or fitness-test measures rather than race performance; sparse and inconsistent evidence; small samples and imprecision.	Very low
Psychological skills training	Competition-related psychological outcomes	Several studies reported favorable changes in negative thoughts, mental toughness, flow, confidence, motivation, or strategy use. Most of these signals came from uncontrolled, single-case, or small quasi-experimental studies, and the favorable pattern became notably thinner when those designs were set aside.	Risk of bias and absence of concurrent controls in several studies; very small samples; heterogeneous psychological constructs; frequent use of bespoke or study-specific measures; limited precision and reduced robustness in sensitivity analysis.	Very low
Mindfulness-based interventions	Competition-related psychological outcomes	Selected outcomes showed a more coherent favorable pattern, particularly refocusing, flow, worry, and cognitive anxiety. The pattern was not uniform: awareness, acceptance, somatic anxiety, dispositional mindfulness, and emotion-regulation outcomes did not consistently improve.	Risk-of-bias concerns, including unblinded self-report; small samples; variation in constructs and instruments; some comparative consistency for selected outcomes, but insufficient breadth or precision for stronger confidence.	Low

GRADE, Grading of Recommendations Assessment, Development and Evaluation.

## 4 Discussion

The findings of this review indicate that the role of psychological preparation in distance running is more nuanced than a simple question of whether mental training improves performance. Across the included studies, PST and mindfulness-based approaches were examined in markedly different competitive settings, from middle-distance and cross-country running to marathon and ultramarathon participation. A more consistent pattern emerged for selected competition-related psychological responses than for objective running outcomes. Improvements were reported in constructs such as flow, attentional refocusing, worry, cognitive anxiety, and negative race-related thoughts, whereas effects on running performance were less consistent and, in several cases, derived from indirect outcomes or methodologically weak designs. The distinction is relevant to contemporary coaching practice because psychological preparation may contribute to how runners regulate attention, emotions, effort, and behavior without necessarily producing an immediate or measurable improvement in race performance. At the same time, confidence in these observations remains limited. Favorable results frequently originated from small samples, uncontrolled or single-subject studies, and self-reported outcomes, while several controlled studies carried substantial risk-of-bias concerns. The current literature therefore supports neither dismissal of psychological approaches nor their routine prescription as established performance-enhancing methods. Rather, it suggests a developing area of athlete preparation in which potentially useful psychological strategies need to be considered alongside the individual runner, competitive context, intended outcome, and strength of the supporting evidence.

### 4.1 Differential patterns across intervention families

Mindfulness-based and acceptance-based studies were concentrated more on self-regulatory, affective, and attentional outcomes than on direct race or time-trial performance. Across this cluster, the most coherent low-certainty signals concerned flow-related constructs, sport-specific refocusing, anxiety or worry, and selected emotion-regulation outcomes. Hill et al. (2021) reported improved flow after mindfulness training in moderately trained runners, and Kelemen et al. (2025) reported improvements in flow and cognitive anxiety in high-level distance runners. De Petrillo et al. (2009) reported improvements in sport-anxiety worry and selected mindfulness- and perfectionism-related variables. Even within this generally favorable psychological pattern, however, effects were not consistent across all constructs. Barbry et al. (2025b) reported a significant benefit for refocusing but not for awareness or acceptance; Hill et al. (2021) did not observe a significant change in dispositional mindfulness; and Kelemen et al. (2025) did not report significant group-by-time effects for somatic anxiety or emotion-regulation difficulties. Accordingly, the mindfulness literature in runners provides only a low-certainty signal for selected psychological targets, rather than robust evidence of broad or reliable improvement across psychological domains.

By contrast, the psychological skills training literature was broader and more fragmented. Psychological skills training encompassed multi-component packages, individualized imagery scripts, self-talk interventions, goal-setting manipulations, relaxation and biofeedback approaches, and hybrid motivational imagery models. This diversity reflects the applied flexibility of psychological skills training, but it also complicates synthesis because studies targeted different mechanisms and outcomes. Jaenes et al. (2021) reported reductions in negative thoughts and mental burden in amateur marathon runners after a 7-weeks psychological skills training program. Koehn and Díaz-Ocejo (2022) reported increased flow in elite junior middle-distance runners following individualized imagery, and runner-specific extracts from Patrick and Hrycaiko (1998) and Cooper et al. (2021) suggested improved running performance after multi-component mental training or personalized strategic self-talk. These favorable findings should be interpreted cautiously because they came mainly from uncontrolled, single-case, or small-sample designs. In more controlled comparisons, McCormick et al. (2018) did not find a significant benefit of motivational self-talk over an active control for ultramarathon finish time, self-efficacy, or perceived control. Weinberg et al. (2012) found no main effects for self-talk type or for assigned versus self-selected self-talk despite some subgroup-level improvements, and Robinson et al. (2023) reported greater improvement in the active-control mental-preparation condition than in the pooled brief-intervention condition. Tenenbaum et al. (1999) similarly found that all goal-setting groups improved over time, but goal difficulty did not differentially affect running performance. Thus, the psychological skills training literature does not support a simple conclusion that one technique or delivery mode is broadly superior across distance-running contexts.

### 4.2 Objective performance outcomes

The running-related findings are more interpretable when direct performance is separated from physiological or fitness indicators and from participation outcomes. Direct running performance was represented by prospectively recorded race or time-trial measures such as 800-m, 1,600-m, one-mile, 2.3-km and 5-km performance, official ultramarathon finishing time, and objectively recorded race-pacing data. These outcomes provide the closest test of whether an intervention changed running performance itself. By contrast, running economy, oxygen consumption, heart rate, ventilation, intermittent maximum speed, shuttle-run performance, and similar standardized fitness or laboratory measures were treated as performance-related indicators. Starting or completing a target ultramarathon was considered a behavioral participation or persistence outcome. These domains may be related, but improvement in one should not be taken as evidence of improvement in another.

Within the PST literature, some of the favorable direct-performance findings came from very small runner-specific single-case data. In Patrick and Hrycaiko (1998) and the synthesis was restricted to the elite 1,500-m runner rather than the three triathletes included in the original study. Similarly, only the trail runner in Cooper et al. (2021) met the runner-specific eligibility criterion. Both participants improved their running times after introduction of the respective mental-skills intervention, but each finding represents one eligible runner and provides limited evidence for generalization. Rhodes et al. (2021) address a related but different question. Participants allocated to Functional Imagery Training were more likely to start and complete an ultramarathon, but start/completion status was treated here as a behavioral participation and persistence outcome rather than direct performance. This distinction matters because completing an event does not establish that running speed, race time, placing, or another performance metric improved. The study may therefore be more informative about commitment to a demanding running goal than about performance enhancement itself. Its interpretation is further limited by the small sample, variation in race distance and conditions, self-directed physical training, and the fact that participants entered the wider project as non-runners.

More controlled comparisons generally provided less consistent evidence for direct performance enhancement. McCormick et al. (2018) found no significant advantage of motivational self-talk over an active control in a real ultramarathon context. De Petrillo et al. (2009) did not demonstrate improved running performance after Mindful Sport Performance Enhancement despite several favorable psychological changes. Weinberg et al. (2012) reported overall pre-post improvement in collegiate cross-country runners, but not a main effect of self-talk type or of assigned versus self-selected self-talk. Tenenbaum et al. (1999) similarly found no differential effect of goal difficulty despite improvement across groups. Robinson et al. (2023) is particularly instructive because the active-control group improved more than the intervention condition, emphasizing the possibility that encouragement to prepare mentally, expectancy effects, or self-regulatory engagement may contribute meaningfully to performance change even in the absence of a formalized psychological technique.

Mindfulness-based interventions did not provide direct race or time-trial evidence. The available running-related findings came instead from physiological or fitness indicators. Hill et al. (2021) reported a within-group reduction in oxygen consumption during running, whereas Barbry et al. (2025a) reported favorable interactions for intermittent maximum speed and the 3-min shuttle-run test. These outcomes may be relevant to running capacity or economy, but they do not demonstrate that mindfulness improved actual race or standardized time-trial performance.

### 4.3 Competition-related psychological outcomes

The psychological-outcome findings should be interpreted primarily as preliminary signals. Favorable changes were reported for flow, worry, refocusing, cognitive anxiety, or negative thoughts in several studies, but the evidential weight of these findings is limited by small samples, self-report measurement, lack of blinding, uncontrolled designs, and heterogeneity in psychological constructs. In this review, favorable findings for flow were observed in Hill et al. (2021), Koehn and Díaz-Ocejo (2022), and Kelemen et al. (2025), although these studies ranged from randomized comparisons to quasi-experimental and single-case evaluations. Similarly, reductions in competition-related worry or maladaptive cognition were reported by De Petrillo et al. (2009) and Jaenes et al. (2021).

One possible explanation for psychological change occurring without a parallel performance effect is that psychological outcomes may respond over a different time course and may be less constrained by physiological capacity, training status, pacing demands, or environmental conditions than race performance. This interpretation remains a hypothesis, however, because the included studies did not formally test whether changes in psychological constructs mediated subsequent changes in performance. The findings by De Petrillo et al. (2009) and Hill et al. (2021) illustrate the distinction: psychological variables changed in some analyses without a corresponding improvement in direct running performance or across all measured constructs. These observations therefore support separation of psychological and performance outcomes rather than a causal explanation linking one to the other.

Acceptability and perceived usefulness should also be considered separately from psychological efficacy. McCormick et al. (2018) found no significant intervention effect on self-efficacy or perceived control, yet 11 of 14 participants in the self-talk group reported that the strategy had been helpful during the ultramarathon, and most respondents continued to use self-talk 6 months later. Such findings are relevant to uptake and sustained use, but they do not demonstrate change in the prespecified psychological constructs or establish efficacy. Conversely, an intervention could produce a measurable psychological effect but still prove difficult to adopt or sustain in routine practice. The limited acceptability information available in the present literature should therefore be interpreted as an implementation consideration alongside, rather than as part of, the efficacy evidence. Cardenas Hernandez et al. (2025) reported descriptive changes in motivation, concentration, commitment, and strategy use, but the uncontrolled design and variation in race contexts preclude strong causal interpretation. Overall, the psychological-outcome evidence remains more consistently favorable than the direct-performance evidence, but its certainty is low.

### 4.4 Population, context, and delivery characteristics

A large proportion of the included evidence came from recreational, amateur, developmental, collegiate, or trained-but-non-elite runners, whereas elite and international-level samples were uncommon and generally small (Patrick and Hrycaiko, 1998; Koehn and Díaz-Ocejo, 2022; Kelemen et al., 2025). No included study was designed to test competitive level as an effect modifier, and no trial reported an intervention-by-competitive-level interaction. The present review therefore cannot determine whether recreational and elite runners respond differently to psychological interventions. Differences in performance constraints, competitive experience, available support, and proximity to the individual performance ceiling provide reasonable grounds for such a hypothesis, but they should not be interpreted as established practice implications. Future studies should recruit sufficiently heterogeneous samples or prespecified competitive-level strata and test these differences directly rather than infer them from separate small studies.

Adolescence encompasses ongoing cognitive and psychosocial development, and the athlete’s autonomy, opportunities for independent psychological-skills practice, and relationships with coaches and parents may influence both intervention delivery and observed outcomes. With only one clearly adolescent, however, the present review cannot determine whether intervention effects differ systematically by developmental stage.

The distribution of disciplines was also wide. Studies covered middle-distance, cross-country, long-distance, marathon, and ultramarathon settings, as well as laboratory-based treadmill tasks that do not map neatly onto field competition. This aspect is a strength from an evidence-mapping perspective, but it complicates direct comparison because the relevant psychological demands differ across events. Ultramarathon studies, for example, more often centered on persistence, coping with prolonged exertion, and completion (McCormick et al., 2018; Rhodes et al., 2021), whereas middle-distance and one-mile studies focused more on pacing, self-talk, and immediate performance execution (Patrick and Hrycaiko, 1998; Weinberg et al., 2012). It is therefore plausible that some of the inconsistency in findings reflects not merely methodological weakness but genuine event-specific differences in how psychological interventions operate.

Another feature was the predominance of repeated, multi-session interventions. Most of the included studies delivered interventions over approximately 4–8 weeks, sometimes with home practice, booster contacts, or repeated laboratory or training exposures. There was little evidence on acute or single-session interventions in genuinely runner-specific samples. Most of the included studies delivered interventions over approximately 4–8 weeks, sometimes with home practice, booster contacts, or repeated laboratory or training exposures. There was little evidence on acute or single-session interventions in genuinely runner-specific samples. This suggests that the current literature implicitly treats psychological change as a process requiring rehearsal and integration into training rather than as an immediate ergogenic effect. However, adherence, completion of independent practice, intervention fidelity, facilitator preparation, and possible contamination or concurrent psychological support were inconsistently reported. Consequently, a null finding cannot always be distinguished clearly from limited intervention exposure or incomplete implementation.

The timing of assessment also limits interpretation. Most studies were better suited to identifying an initial post-intervention response than to establishing whether psychological or performance changes persisted after continued training or repeated competition exposure. This distinction may be especially relevant for interventions that depend on rehearsal and application of a skill over time. Conversely, state-like outcomes assessed during or shortly after a running task may capture relatively immediate responses that need not persist. The small amount of later follow-up therefore prevents firm conclusions about maintenance or consolidation of the reported effects.

Delivery formats varied from individualized face-to-face sessions and workbook-based programmes to online group delivery, prerecorded online material, and home-based digital training. Online delivery has therefore been used successfully as a mode of intervention delivery in runner samples, including the 6-weeks group-based MSPE programme of Kelemen et al. (2025) and the brief online interventions evaluated by Robinson et al. (2023). However, these studies were not designed as formal feasibility evaluations, and reporting of recruitment yield, retention, technical burden, participant burden, acceptability, and implementation success was incomplete. The present evidence therefore shows that remote delivery is possible, but it does not establish its feasibility relative to face-to-face provision or determine whether one delivery mode produces better adherence, acceptability, or outcomes.

Acceptability was also reported inconsistently. Some studies included participant feedback, satisfaction ratings, perceived usefulness, or continued strategy use, but these outcomes were neither defined nor measured consistently enough for comparative synthesis. Where available, they are better understood as indicators of whether participants were willing to engage with or continue using an intervention rather than evidence that the intervention changed its intended psychological or performance target. Future trials should prespecify acceptability alongside adherence and fidelity when implementation in routine athlete-support settings is an intended aim.

### 4.5 Methodological limitations of the evidence and research gaps

Several recurring methodological weaknesses limit certainty across the review. Many studies were small, and some were extremely small, including single-subject or runner-specific extracts from mixed-sport samples (Patrick and Hrycaiko, 1998; Cooper et al., 2021). Small samples increase the risk of unstable estimates, selective interpretation of isolated positive findings, and inadequate control for baseline differences. Moreover, the evidence base included a substantial number of quasi-experimental studies without robust concurrent controls, which makes it difficult to separate intervention effects from training progression, seasonal change, expectancy, or regression to the mean. Even among randomized studies, comparator conditions varied considerably, and active-control conditions were uncommon. Where active controls were used, they sometimes performed as well as or better than the focal intervention (McCormick et al., 2018; Robinson et al., 2023), emphasizing the importance of controlling for attention, expectancy, and self-regulatory engagement.

A further limitation is that the available studies did not permit a defensible meta-analysis. The evidence base combined randomized trials, non-randomized controlled studies, single-group studies, and single-subject designs, with heterogeneous runner populations, interventions, comparators, and outcome definitions. Consequently, the review emphasizes structured narrative synthesis rather than pooled effect estimation, and the conclusions should be interpreted as evidence mapping and qualitative integration rather than as quantitative estimates of intervention efficacy.

The certainty-of-evidence assessment further indicates that the conclusions of this review should be interpreted cautiously. No intervention-family/outcome-domain combination was supported by moderate- or high-certainty evidence. Certainty was downgraded because of frequent risk-of-bias concerns, small samples, heterogeneity in populations and outcome definitions, limited use of active comparators, inconsistent performance findings, and indirectness where outcomes were laboratory or fitness-test measures rather than direct race-performance endpoints. Therefore, the review supports evidence mapping and cautious interpretation of possible psychological benefits, but it does not provide high-certainty evidence of performance enhancement.

A search-related limitation is that SPORTDiscus and PsycINFO were not directly searched. To reduce the likelihood of missing eligible studies, we supplemented the database searches with backward citation searching of included reports and related reviews, forward citation searching in Scopus and Web of Science, and specific author/intervention searches. Nevertheless, the possibility that some eligible records indexed only in unavailable discipline-specific databases were missed cannot be excluded.

The most important research gap is the absence of a coherent, adequately powered runner-specific trial literature linking clearly defined psychological targets to meaningful running outcomes. Future trials should use preregistration, adequate sample sizes, explicit allocation procedures, and active-control conditions that account for expectancy and contact time. Direct running performance, physiological or fitness indicators, participation outcomes, psychological efficacy outcomes, and acceptability or implementation outcomes should be defined separately before analysis. Where a psychological construct is proposed as a mechanism through which performance may change, mediation should be tested prospectively rather than inferred from parallel changes in the two outcomes.

Greater attention is also needed to event type and competitive level. Middle-distance, cross-country, road, marathon, and ultramarathon running present different task demands, and elite runners remain markedly underrepresented. Future studies should therefore prespecify competitive level and, where sample size permits, examine intervention-by-level interactions or stratified effects rather than assuming that findings from recreational runners generalize to high-performance athletes. The same principle applies to developmental stage.

Longer-term follow-up should become a routine part of this research. Most current studies were better suited to detecting an initial post-intervention response than to establishing persistence. Future trials should test whether changes in attention, anxiety, mindfulness, flow, coping, or other targeted processes are maintained after the intervention has ended and across subsequent training or competition exposure. They should then examine whether any sustained psychological change translates into meaningful behavioral, training, participation, or direct running-performance outcomes. This would help distinguish a transient state response from a durable change with practical relevance.

Implementation should be evaluated alongside efficacy. Recruitment, retention, attendance, home-practice completion, fidelity, practitioner characteristics, acceptability, technical burden, and unintended effects should be reported prospectively. This would make it possible to distinguish an intervention that fails because it does not affect the intended target from one that is difficult to deliver or insufficiently used, and to identify approaches that athletes are willing to sustain even when immediate efficacy is uncertain.

### 4.6 Provisional considerations for coaching practice

The following considerations for coaching practice are necessarily provisional. None of the included studies was designed to test a coaching model or to establish the effectiveness of psychological interventions when implemented routinely by coaches, and no intervention-family/outcome-domain combination was supported by moderate- or high-certainty evidence. The implications below are therefore derived from the pattern of runner-specific intervention findings, intervention characteristics, implementation information, and participant contexts rather than from direct tests of coaching practice. Within that limitation, PST and mindfulness-based approaches should not be treated as interchangeable techniques or as standardized additions expected to improve performance in every runner. The available evidence is more consistent with using selected strategies to address a clearly identified psychological or self-regulatory target, such as competition-related worry, attentional control, coping with discomfort, or task-specific flow. Whether change in that target subsequently affects running performance remains uncertain and should be evaluated rather than assumed.

This interpretation is compatible with an athlete-centered approach to coaching, in which psychological strategies are selected in relation to the runner’s experiences, competitive demands, preferences, and response to previous practice rather than prescribed solely because a particular technique has been associated with favorable outcomes elsewhere. The considerable variation across running disciplines in the present review reinforces this point. The psychological demands associated with an 800-m or 1,500-m race are not identical to those encountered during a marathon or ultramarathon, and the relevance of a given intervention may differ across these contexts, although this has not been tested directly. Where PST or mindfulness-based methods are incorporated into training, coaches and sport-psychology practitioners should therefore define the intended target in advance and evaluate whether change occurs in that target rather than assuming that psychological improvement will automatically transfer to faster running performance.

A further implication concerns how psychological support is embedded within the broader coaching environment. Coaches may reinforce performance-focused psychological skills that fall within their training and professional competence, whereas structured psychological programmes, clinically significant distress, or concerns requiring assessment or treatment should involve appropriately qualified practitioners and clear referral pathways (Carr, 2006; Bissett et al., 2020). Rather than positioning mental training as an isolated component of preparation, coaches may have greater practical value in coordinating psychological strategies with training demands, competition planning, pacing, recovery, and athlete feedback. Such integration should remain appropriately individualized and, where specialist psychological expertise is required, interdisciplinary. Monitoring is also important. Psychological outcomes and objective performance indicators should be considered separately because the current evidence shows that they can follow different patterns of response. A runner may experience better attentional control, reduced worry, or greater flow without a detectable improvement in race time, and such a result should not necessarily be regarded as either intervention failure or evidence of performance enhancement.

These considerations should remain proportionate to the certainty of the evidence. PST and mindfulness-based approaches cannot currently be presented as established methods for improving distance-running performance. Where they are used, a defensible approach is to treat them as individualized adjuncts to wider athlete preparation, with an explicit target, appropriate professional oversight, and monitoring of psychological response, adherence, acceptability, and relevant running outcomes. Future applied studies should test these coaching-context assumptions directly, including how interventions are incorporated into normal training, who delivers them, which runners engage with them, and whether sustained psychological changes contribute to meaningful training or competition outcomes.

## 5 Conclusion

Runner-specific evidence for psychological skills training and mindfulness-based interventions remains limited in both quantity and certainty. The clearest findings concern selected competition-related psychological outcomes, including flow, refocusing, worry, cognitive anxiety, and negative race-related thoughts. Evidence for direct improvement in running performance is less consistent, and some apparently favorable findings arise from small, uncontrolled, or single-case studies. Physiological or fitness-related signals and event participation outcomes should not be interpreted as equivalent to faster race or time-trial performance. Acceptability and perceived usefulness form a separate part of the evidence. Some runners reported finding particular strategies useful or continued to use them after the study period, but such observations indicate potential uptake or sustained use rather than efficacy. Similarly, the available studies show that psychological interventions can be delivered through face-to-face, workbook, online, and digital formats, but they do not establish the comparative feasibility or effectiveness of those delivery modes. For coaching practice, the current evidence supports only provisional considerations. Psychological strategies may be considered as individualized adjuncts when a specific competition-related or self-regulatory need has been identified, but they should not be presented as reliably performance-enhancing techniques. Differences according to event type, competitive level, developmental stage, intervention dose, or delivery mode remain hypotheses requiring direct testing. Future runner-specific trials should combine stronger controlled designs with explicit implementation and acceptability outcomes, competitive-level stratification where possible, and longer-term follow-up to determine whether psychological changes persist and whether they subsequently translate into meaningful training, participation, or competition outcomes.

## Data Availability

The original contributions presented in this study are included in this article/supplementary material, further inquiries can be directed to the corresponding author.

## References

[B1] Barbry A. Carton A. Bernier M. Ovigneur H. Coquart J. (2025a). Does brief mindfulness intervention increase performance? An exploratory study. *Int. J. Sports Med*. 46, 690–700. 10.1055/a-2544-7523 39986265

[B2] Barbry A. Coquart J. Bernier M. Daubresse A. Ovigneur H. Carton A. (2025b). An exploratory randomized controlled study on the effects of a brief mindfulness intervention with high-intensity training on mindfulness skills and well-being in runners. *Mindfulness* 16, 3535–3551. 10.1007/s12671-025-02713-6

[B3] Barker T. H. Habibi N. Aromataris E. Stone J. C. Leonardi-Bee J. Sears K. et al. (2024). The revised JBI critical appraisal tool for the assessment of risk of bias for quasi-experimental studies. *JBI Evid. Synth*. 22, 378–388. 10.11124/JBIES-23-00268 38287725

[B4] Basset F. A. Kelly L. P. Hohl R. Kaushal N. (2022). Type of self-talk matters: Its effects on perceived exertion, cardiorespiratory, and cortisol responses during an iso-metabolic endurance exercise. *Psychophysiology* 59:e13980. 10.1111/psyp.13980 34837395

[B5] Bengtsson D. Stenling A. Nygren J. Ntoumanis N. Ivarsson A. (2024). The effects of interpersonal development programmes with sport coaches and parents on youth athlete outcomes: A systematic review and meta-analysis. *Psychol. Sport Exerc*. 70:102558. 10.1016/j.psychsport.2023.102558 37993028

[B6] Birrer D. Morgan G. (2010). Psychological skills training as a way to enhance an athlete’s performance in high-intensity sports. *Scand. J. Med. Sci. Sports*. 20, 78–87. 10.1111/j.1600-0838.2010.01188.x 20840565

[B7] Bissett J. E. Kroshus E. Hebard S. (2020). Determining the role of sport coaches in promoting athlete mental health: A narrative review and delphi approach. *BMJ Open Sport Exerc. Med*. 6:e000676. 10.1136/bmjsem-2019-000676 32095271 PMC7011013

[B8] Brown D. J. Fletcher D. (2017). Effects of psychological and psychosocial interventions on sport performance: A meta-analysis. *Sports Med*. 47, 77–99. 10.1007/s40279-016-0552-7 27241124

[B9] Bühlmayer L. Birrer D. Röthlin P. Faude O. Donath L. (2017). Effects of mindfulness practice on performance-relevant parameters and performance outcomes in sports: A meta-analytical review. *Sports Med*. 47, 2309–2321. 10.1007/s40279-017-0752-9 28664327

[B10] Caird S. J. McKenzie A. D. Sleivert G. G. (1999). Biofeedback and relaxation techniques improves running economy in sub-elite long distance runners. *Med. Sci. Sports Exerc*. 31, 717–722. 10.1097/00005768-199905000-00015 10331893

[B11] Campbell M. McKenzie J. E. Sowden A. Katikireddi S. V. Brennan S. E. Ellis S. et al. (2020). Synthesis without meta-analysis (SWiM) in systematic reviews: Reporting guideline. *BMJ*. 368:l6890. 10.1136/bmj.l6890 31948937 PMC7190266

[B12] Cardenas Hernandez F. P. Schneider J. Nagler T. Parada-Cabaleiro E. Schiffner D. Dengel A. et al. (2025). Miles with mind: An exploratory study of cognitive-behavioral-based VR training for strategy and motivation in long-distance running. *Front. Sports Act. Living*. 7:1722120. 10.3389/fspor.2025.1722120 41393315 PMC12698523

[B13] Carr C. M. (2006). Sport psychology: Psychologic issues and applications. *Phys. Med. Rehabil. Clin. Am*. 17, 519–535. 10.1016/j.pmr.2006.05.007 16952750

[B14] Casado A. Hanley B. Jiménez-Reyes P. Renfree A. (2021). Pacing profiles and tactical behaviors of elite runners. *J. Sport Health Sci*. 10, 537–549. 10.1016/j.jshs.2020.06.011 32599344 PMC8500812

[B15] Cooper K. B. Wilson M. R. Jones M. I. (2021). Fast talkers? Investigating the influence of self-talk on mental toughness and finish times in 800-meter runners. *J. Appl. Sport Psychol.* 33, 491–509. 10.1080/10413200.2020.1735574

[B16] Corbally L. Wilkinson M. Fothergill M. A. (2020). Effects of mindfulness practice on performance and factors related to performance in long-distance running: A systematic review. *J. Clin. Sport Psychol.* 14, 376–398. 10.1123/jcsp.2019-0034

[B17] De Petrillo L. A. Kaufman K. A. Glass C. R. Arnkoff D. B. (2009). Mindfulness for long-distance runners: An open trial using mindful sport performance enhancement (MSPE). *J. Clin. Sport Psychol.* 3, 357–376. 10.1123/jcsp.3.4.357

[B18] Dehghani M. Saf A. D. Vosoughi A. Tebbenouri G. Zarnagh H. G. (2018). Effectiveness of the mindfulness-acceptance-commitment-based approach on athletic performance and sports competition anxiety: A randomized clinical trial. *Electron Physician*. 10, 6749–6755. 10.19082/6749 29997757 PMC6033134

[B19] Donohue B. Miller A. Beisecker M. Houser D. Valdez R. Tiller S. et al. (2006). Effects of brief yoga exercises and motivational preparatory interventions in distance runners: Results of a controlled trial. *Br. J. Sports Med.* 40, 60–63. 10.1136/bjsm.2005.020024 16371493 PMC2491926

[B20] Gardner F. L. Moore Z. E. (2017). Mindfulness-based and acceptance-based interventions in sport and performance contexts. *Curr. Opin. Psychol*. 16, 180–184. 10.1016/j.copsyc.2017.06.001 28813347

[B21] Goddard S. G. Stevens C. J. Swann C. (2024). A flow intervention for runners: Mixed-method evaluation. *Sport Exerc. Perform. Psychol.* 13, 144–161. 10.1037/spy0000340

[B22] Grandjean da Costa K. Urry H. L. Fontes E. B. Elliott G. Cantelon J. A. Giles G. E. (2022). Cognitive reappraisal mitigates affective valence declines during exercise at the ventilatory threshold. *Int. J. Sport Exerc. Psychol.* 20, 1471–1489. 10.1080/1612197X.2021.1979080

[B23] Hill A. Schücker L. Wiese M. Hagemann N. Strauß B. (2021). The influence of mindfulness training on running economy and perceived flow under different attentional focus conditions – an intervention study. *Int. J. Sport Exerc. Psychol.* 19, 564–583. 10.1080/1612197X.2020.1739110

[B24] Jackson S. A. (1996). Toward a conceptual understanding of the flow experience in elite athletes. *Res. Q. Exerc. Sport*. 67, 76–90. 10.1080/02701367.1996.10607928 8735997

[B25] Jaenes J. C. Wilczyńska D. Alarcón D. Peñaloza R. Casado A. Trujillo M. (2021). The effectiveness of the psychological intervention in amateur male marathon runners. *Front. Psychol*. 12:605130. 10.3389/fpsyg.2021.605130 33841241 PMC8024526

[B26] Kelemen B. Tóth R. Benczenleitner O. Tóth L. (2025). Effects of group mindfulness intervention on high-level distance runners: A quasi-experimental study. *Front. Sports Act. Living* 7:1556404. 10.3389/fspor.2025.1556404 40336708 PMC12055820

[B27] Koehn S. Díaz-Ocejo J. (2022). Imagery intervention to increase flow state: A single-case study with middle-distance runners in the state of Qatar. *Int. J. Sport Exerc. Psychol.* 20, 729–742. 10.1080/1612197X.2016.1187653

[B28] Mageau G. A. Vallerand R. J. (2003). The coach-athlete relationship: A motivational model. *J Sports Sci*. 21, 883–904. 10.1080/0264041031000140374 14626368

[B29] Marcora S. M. Staiano W. Manning V. (2009). Mental fatigue impairs physical performance in humans. *J. Appl .Physiol*. 106, 857–864. 10.1152/japplphysiol.91324.2008 19131473

[B30] McCormick A. Meijen C. Marcora S. (2015). Psychological determinants of whole-body endurance performance. *Sports Med*. 45, 997–1015. 10.1007/s40279-015-0319-6 25771784 PMC4473096

[B31] McCormick A. Meijen C. Marcora S. (2018). Effects of a motivational self-talk intervention for endurance athletes completing an ultramarathon. *Sport Psychol.* 32, 42–50. 10.1123/tsp.2017-0018

[B32] McKay D. Broderick C. Steinbeck K. (2016). The adolescent athlete: A developmental approach to injury risk. *Pediatr. Exerc. Sci*. 28, 488–500. 10.1123/pes.2016-0021 27705538

[B33] Midgley A. W. McNaughton L. R. Jones A. M. (2007). Training to enhance the physiological determinants of long-distance running performance: Ean valid recommendations be given to runners and coaches based on current scientific knowledge? *Sports Med*. 37, 857–880. 10.2165/00007256-200737100-00003 17887811

[B34] Page M. J. McKenzie J. E. Bossuyt P. M. Boutron I. Hoffmann T. C. Mulrow C. D. et al. (2021). The PRISMA 2020 statement: an updated guideline for reporting systematic reviews. *BMJ*. 372:n71. 10.1136/bmj.n71 33782057 PMC8005924

[B35] Pageaux B. Lepers R. (2016). Fatigue induced by physical and mental exertion increases perception of effort and impairs subsequent endurance performance. *Front. Physiol*. 7:587. 10.3389/fphys.2016.00587 27965592 PMC5126404

[B36] Patrick T. D. Hrycaiko D. W. (1998). Effects of a mental training package on an endurance performance. *Sport Psychol.* 12, 283–299. 10.1123/tsp.12.3.283

[B37] Raabe J. Schmidt K. Carl J. Höner O. (2019). The effectiveness of autonomy support interventions with physical education teachers and youth sport coaches: a systematic review. *J Sport Exerc Psychol*. 41, 345–355. 10.1123/jsep.2019-0026 31722291

[B38] Reinebo G. Alfonsson S. Jansson-Fröjmark M. Rozental A. Lundgren T. (2024). Effects of psychological interventions to enhance athletic performance: a systematic review and meta-analysis. *Sports Med*. 54, 347–373. 10.1007/s40279-023-01931-z 37812334 PMC10933186

[B39] Rethlefsen M. L. Kirtley S. Waffenschmidt S. Ayala A. P. Moher D. Page M. J. et al. (2021). PRISMA-S: an extension to the prisma statement for reporting literature searches in systematic reviews. *Syst Rev*. 10:39. 10.1186/s13643-020-01542-z 33499930 PMC7839230

[B40] Rhodes J. Nedza K. May J. Jenkins T. Stone T. (2021). From couch to ultra marathon: using functional imagery training to enhance motivation. *J. Imag. Res. Sport Physical Act.* 16. 10.1515/jirspa-2021-0011

[B41] Robinson D. T. Holliday S. W. Cloak R. Lane A. M. (2023). Brief Online Interventions to Improve 5K Running. *J. Coach. Sport. Sci.* 2, 24–33. 10.58524/jcss.v2i1.214

[B42] Röthlin P. Horvath S. Trösch S. Holtforth M. G. Birrer D. (2020). Differential and shared effects of psychological skills training and mindfulness training on performance-relevant psychological factors in sport: a randomized controlled trial. *BMC Psychol*. 8:80. 10.1186/s40359-020-00449-7 32762736 PMC7409666

[B43] Samadi H. Maleki B. Sohbatiha M. (2020). The effectiveness of mindfulness-based cognitive-behavioral strategy training on cognitive emotion regulation strategies and salivary cortisol levels in endurance runners: a three-month follow-up. *Middle East J. Rehab. Health Stud.* 7. 10.5812/mejrh.100953

[B44] Sha J. Yi Q. Jiang X. Wang Z. Cao H. Jiang S. (2024). Pacing strategies in marathons: A systematic review. *Heliyon* 10:e36760. 10.1016/j.heliyon.2024.e36760 39281580 PMC11400961

[B45] Sterne J. A. C. Savović J. Page M. J. Elbers R. G. Blencowe N. S. Boutron I. et al. (2019). RoB 2: a revised tool for assessing risk of bias in randomised trials. *BMJ*. 366:l4898. 10.1136/bmj.l4898 31462531

[B46] Sterne J. A. Hernán M. A. Reeves B. C. Savović J. Berkman N. D. Viswanathan M. et al. (2016). ROBINS-I: a tool for assessing risk of bias in non-randomised studies of interventions. *BMJ*. 355:i4919. 10.1136/bmj.i4919 27733354 PMC5062054

[B47] Tanay G. Bernstein A. (2013). State mindfulness scale (SMS): development and initial validation. *Psychol. Assess*. 25, 1286–1299. 10.1037/a0034044 24059475

[B48] Tenenbaum G. Spence R. Christensen S. (1999). The effect of goal difficulty and goal orientation on running performance in young female athletes. *Aust. J. Psychol.* 51, 6–11. 10.1080/00049539908255328

[B49] Thompson R. W. Kaufman K. A. De Petrillo L. A. Glass C. R. Arnkoff D. B. (2011). One year follow-up of mindful sport performance enhancement (MSPE) with archers, golfers, and runners. *J. Clin. Sport Psychol.* 5, 99–116. 10.1123/jcsp.5.2.99

[B50] Wang Y. Lei S. M. Fan J. (2023). Effects of mindfulness-based interventions on promoting athletic performance and related factors among athletes: A systematic review and meta-analysis of randomized controlled trial. *Int. J. Environ. Res. Public Health*. 20:2038. 10.3390/ijerph20032038 36767403 PMC9915077

[B51] Weinberg R. Miller A. Horn T. (2012). The influence of a self-talk intervention on collegiate cross-country runners. *Int. J. Sport Exerc. Psychol.* 10, 123–134. 10.1080/1612197X.2012.645135

[B52] Xie B. Lei S. Choi N. Choi S. M. Wang X. Chen Y. (2025). Impact of mindfulness-based interventions on sports performance and mental health: An umbrella review. *J. Exerc. Sci. Fit*. 23, 261–272. 10.1016/j.jesf.2025.06.008 40689310 PMC12273557

